# Supplementary material for: Protocol for a mixed studies systematic review on the implementation of the recovery approach in adult mental health services
Source: BMJ Open. 2017 Aug 30;7(8):e017080. doi: 10.1136/bmjopen-2017-017080 (PMC5724147; doi:10.1136/bmjopen-2017-017080)
Supplement: Supplementary file 2 [file bmjopen-2017-017080supp002.pdf]

APPENDIX 2

Pilot search strategy for Ovid MEDLINE(R) In-Process & Other Non-Indexed Citations, Ovid MEDLINE(R) Daily, Ovid MEDLINE(R) and Ovid OLDMEDLINE(R) 1946 to Present

#	Searches
1	recovery oriented.ti,ab,kw.
2	exp Mental Health Services/
3	recover*.ti,ab,kw.
4	2 and 3
5	Mental Health/
6	mental disorders/ or adjustment disorders/ or exp anxiety disorders/ or exp delirium, dementia, amnestic, cognitive disorders/ or exp dissociative disorders/ or exp mood disorders/ or exp neurotic disorders/ or exp personality disorders/ or exp "schizophrenia and disorders with psychotic features"/
7	((Mental or psychiatric or psychologic*) adj2 (health or illness* or disorder* or disease* or problem* or issue or issues or well being)).ti,ab,kw.
8	((adjustment or anxiety or obsessive or complusive or panic or phobic or stress or dissociative or mood or depressive or affective or personality or psychotic or paranoid) adj2 (disorder* or disease*)).ti,ab,kw.
9	(agoraphobi* or astheni* or phobia* or delirium or dementia* or depression or schizophreni* or schizoid).ti,ab,kw.
10	5 or 6 or 7 or 8 or 9
11	exp health services/ or service*.ti,ab,kw.
12	10 and 11
13	exp "Outcome and Process Assessment (Health Care)"/
14	exp delivery of health care/
15	Resource Allocation/
16	exp "Organization and Administration"/
17	exp "Quality of Health Care"/
18	exp professional competence/
19	exp Inservice Training/
20	exp patient care management/
21	((service* or organization* or organisation* or unit or units or department* or program* or

clinic or clinics or ward or wards or staff or workforce or setting) adj4 (leader* or innovate* or objective* or change* or transform* or structure* or opportunit* or strength* or model* or priorit* or policy or policies or procedure* or ration* or allocat* or reform* or commit* or needs or improve* or assess* or responsive* or evaluat* or plan*)).ti,ab,kw.

22 ((treatment or therapy) adj2 planning).ti,ab,kw.

23 13 or 14 or 15 or 16 or 17 or 18 or 19 or 20 or 21 or 22

24 exp Patient-Centered Care/

25 exp Self Care/ or exp Self Concept/

26 ((self or personal) adj2 (identity or concept or assess* or manage* or disclos* or efficacy or evaluat*)).ti,ab,kw.

27 ((patient or client or consumer*) adj4 (centered or centred or focus* or engage* or involve*)).ti,ab,kw.

28 ((shared or joint) adj2 decision*).ti,ab,kw.

29 exp Interpersonal Relations/

30 "Quality of Life"/ or quality of life.mp.

31 Social Support/ or Social Adjustment/

32 (social adj2 (support or integration or inclusion or adjustment)).ti,ab,kw.

33 (connected* or hope* or optimis* or trust or identity or meaning or empower* or spiritual* or giving back or stigma* or values or goal or goals or belonging or purpose or choice or citizenship or discriminat* or inequalit* or valued or roles).ti,ab,kw.

34 (partnership* or collaborat* or interpersonal or multidisciplinary or cooperat* or co-operat* or team* or group or joint).ti,ab,kw.

35 24 or 25 or 26 or 27 or 28 or 29 or 30 or 31 or 32 or 33 or 34

36 exp Data Collection/

37 exp Psychological Tests/

38 (measur* or test* or scale* or metric* or psychometric* oe questionnaire* or instrument* or data).ti,ab,kw.

39 36 or 37 or 38

40 23 or 39

41 12 and 40

42 41 and recover*.mp.

43 1 or 4 or 42
