# Supplementary material for: Protocol for a mixed studies systematic review on the implementation of the recovery approach in adult mental health services
Source: BMJ Open. 2017 Aug 30;7(8):e017080. doi: 10.1136/bmjopen-2017-017080 (PMC5724147; doi:10.1136/bmjopen-2017-017080)
Supplement: Author's manuscript [file bmjopen-2017-017080.draft_revisions.pdf]

BMJ Open

Implementation of the Recovery Concept Into Canadian Mental Health Services: A Protocol For A Mixed Studies Systematic Review

Journal:	*BMJ Open*
Manuscript ID	bmjopen-2017-017080
Article Type:	Protocol
Date Submitted by the Author:	17-Apr-2017

Complete List of Authors:	Piat, Myra; McGill University, Psychiatry; Douglas Mental Health University Institute,<br>Sofouli, Eleni; Douglas Mental Health University Institute<br>Sabetti, Judith; McGill University, School of Social Work<br>Chodos, Howard; Mental Health Commission of Canada - Ottawa<br>Briand, Catherine; Inst Univ Sante Mentale Montreal,<br>Vachon, Brigitte ; Universite de Montreal Faculte de medecine<br>Curran, Janet; Dalhousie University - Faculty of Health Professions

**Primary Subject Heading**:	Mental health
Secondary Subject Heading:	Evidence based practice, Health services research
Keywords:	MENTAL HEALTH, Adult psychiatry < PSYCHIATRY, Recovery, HEALTH SERVICES

SCHOLARONE™
Manuscripts

Implementation Of The Recovery Concept Into Canadian Mental Health Services: A Protocol For A Mixed Studies Systematic Review

For peer review only

Myra Piat[1,2,3] ,PhD,
Eleni Sofouli[3] ,MSW,
Judith Sabetti[2,3] ,PhD,
Howard Chodos, PhD
Catherine Briand [4], PhD,
Brigitte Vachon [4], PhD,
Janet Curran, PhD,

Corresponding author: Myra Piat, PhD, myra.piat@douglas.mcgill.ca

Douglas Mental Health Institute, 6875 Lasalle Blvd, Montreal, Quebec H4H 1R3

Tel: 647 323-9038 fax: 514 888-4084

Word Count 2804

Author Affiliations:

[1]Department of Psychiatry, McGill University, Montreal, QC, Canada
[2]School of Social Work, McGill University, Montreal, QC, Canada
[3]Douglas Mental Health University Institute, Research Center, Montreal, QC, Canada
[4] Former Director of Mental Health Strategy, Mental Health Commission of Canada
[5]Faculty of Medicine, University of Montreal, Montreal, QC, Canada

[6] School of Nursing, Faculty of Health Professions, Dalhousie University, Halifax, NS, Canada

For peer review only - http://bmjopen.bmj.com/site/about/guidelines.xhtml

ABSTRACT

Introduction: Recovery is integral to mental health planning in G-8 countries including Canada. A recovery-oriented approach to care aims to promote personal empowerment, illness self-management, and a life beyond services for people with serious mental illness (SMI), while reducing the staggering financial burden associated with mental illness. Although there is a growing body of literature on recovery-oriented services, no synthesis of research on the implementation of recovery into mental health services exists.

Objectives: A mixed studies systematic review will be conducted on recovery-oriented services for adults with SMI. Seven databases including PubMed, Ovid Medline, Ovid Embase, Ovid PsycInfo, CINAHL (Current Index to Nursing and Allied Health Literature), the Cochrane Library and Scopus will be searched for peer-reviewed empirical studies published from 1998 to December 2016.

Methods: Systematic reviews, and studies using quantitative, qualitative and mixed methodologies will be included. Secondary searches will be conducted in reference lists of all selected full text articles; and citation-tracking will be performed on articles related to those included in the review. Hand searches will also be performed in the tables of contents of three recovery-focused journals for the most recent 5 years. International experts in the field will be contacted for comments and advice. Data extraction will include identification and methodological synthesis of each study; definition of recovery; information on recovery implementation; facilitators and barriers; and study outcomes. A quality assessment will be conducted on each study. The data will be synthesized and a stepwise thematic analysis performed.

Strengths and limitations of this study

- Studies included in this knowledge synthesis will have been conducted in inpatient, outpatient and community-based mental health settings, and will cover a broad range of research methodologies. The synthesis will reveal how recovery is understood; challenges involved in implementation; and, overall, to what extent transformation to recovery oriented services and systems is occurring.
- The selection of recovery-oriented studies with an implementation focus is unique, and will allow us to draw on 5 powerful conceptual models from implementation science that provide theory-informed elements to guide data analysis and synthesis as well as the reporting of results.

For peer review only - http://bmjopen.bmj.com/site/about/guidelines.xhtml

- Knowledge from the synthesis will be compiled into comprehensive and usable formats for organizational and government stakeholders, providing practical guidelines for recovery-based service reform and future evaluation.
- Limiting the search to published, peer-reviewed studies, while important for considerations of quality and methodological rigor, may overlook possible research on recovery-oriented services reported elsewhere.

Key words : mental health service, recovery-orientation, mental disorders, implementation, protocol, systematic review

For peer review only

Ethics and dissemination:

Ethics approval is not required for this knowledge synthesis. Findings will be disseminated through multiple knowledge translation activities including: a) symposiums; b) presentations in national and international conferences, and to local stakeholders; c) publications in peer-reviewed open access journals, d) posts on the organizational websites of participating organizations.

INTRODUCTION

Rationale

For peer review only - http://bmjopen.bmj.com/site/about/guidelines.xhtml

Recovery is the focus of national mental health plans in G-8 countries[1-3] including Canada's first mental health strategy, *Changing Directions, Changing Lives*[4], and several provincial strategies[5-8]. The rationale for transformation to recovery-oriented services in mental health is compelling. While traditional mental health services have underlined professional control[9-13], reinforcing patient dependency, self-stigma and hopelessness[14-17], recovery approaches focus on individual empowerment, strong collaborative relationships between mental health service providers and service users, and community integration[18-24]. In promoting a life beyond services, recovery also meets a key ethical obligation to honor the personhood and citizenship of people with mental illness[25].

Recovery knowledge and evidence have burgeoned over the past two decades. Research exists on personal recovery[26-32], recovery-oriented services[33-40], and provider competencies[41-45]. Conceptual frameworks and standardized measures have been produced[35,46-49]. Other studies have linked the recovery approach to recognized theories, such as empowerment theory[50,51], the strengths model[52], capabilities theory[53-55], positive psychology[56-58], person-centered practice[59,60] and co-production[61,62]. Practice guidelines for recovery-oriented service provision are available[63-69], as well. In terms of the empirical literature, studies on particular agencies and programs have identified potential determinants of recovery orientation in services: for example a flexible and innovative organizational culture, results-oriented leadership and larger budgets[70] were found to be associated with recovery-oriented services, as were provider socio-professional characteristics such as greater age, higher educational levels and more professional experience[71,72]. Another recent study found

For peer review only - http://bmjopen.bmj.com/site/about/guidelines.xhtml

that increasing the recovery orientation of teamwork on mental health teams[73] was associated with provider and consumer perceptions that services were recovery-oriented.

While two recent systematic reviews have been conducted on the recovery-oriented practices of mental health service providers[74,75], no known review has been published, to date, on implementation of the recovery concept into mental health services. Our review synthesizes research on the nature of recovery-oriented services, implementation challenges and overall system transformation. Work on the project initiated in August 2016, and should come to completion in spring 2018.

This project is important and timely, as mental illness affects millions of people worldwide. According to recent World Health Organization statistics[76], 350 million people are impacted by depression; 60 million people by bipolar disorder, and 21 million affected by schizophrenia. A recovery-oriented approach to healthcare is expected to reduce dependency and reduce the cost of mental healthcare, which in Canada has risen to more than $50 billion per year[4,77,78]. The project responds to a critical knowledge gap identified by knowledge users across Canada, who are responsible for implementing provincial level policy as well as shifting organizational practices to recovery.

Objectives

The goal of this paper is to present the design for a systematic review that will evaluate and synthesize published literature on the implementation of the recovery approach into mental health services for adults with SMI (schizophrenia, bipolar disorder, major depression). Each selected study will be assessed in terms of the following questions: (a) How is recovery defined in this study?; (b) How is the recovery approach implemented in this study? (c) What

For peer review only - http://bmjopen.bmj.com/site/about/guidelines.xhtml

facilitators and barriers emerged in implementing recovery in this study? (d) What outcomes are reported in this study?

METHODS

Eligibility criteria

The design and methodology for the present review are reported following the Preferred Reporting Items for Systematic review and Meta-Analysis Protocols (PRISMA-P) guidelines[79].

Population: The review concerns studies of services for adults (18 yrs. +) with a primary diagnosis of schizophrenia, bipolar disorder or major depression, following the DSM-V classification for mental disorders.

Intervention: Studies will be included if they describe and evaluate the implementation of any intervention based on recovery principles that aims at transforming the orientation of mental health services, or organizations to a recovery approach.

Comparators: Studies will be eligible for inclusion whether or not they include comparison groups.

Outcomes: Selected studies should report outcomes related to the transformation of a mental health service, or organization, to a recovery-orientation. Outcomes might include change in organizational culture; more integrated service networks and partnerships; increased knowledge, skills and/or attitudinal change among mental health providers; more use of evidence based recovery-oriented best practices; greater consumer/provider collaboration, consumer self-management, and evaluation.

Study design: This will be a mixed studies review (MSR)[80,81]. The MSR integrates qualitative, quantitative and mixed methods studies, providing a rich, detailed understanding of complex

For peer review only - http://bmjopen.bmj.com/site/about/guidelines.xhtml

health interventions and programs[82]. Studies representing a full range of methodologies will be included: systematic reviews and meta-analyses, randomized control trials (RCTs) and clinical trials, observational, mixed method and qualitative studies.

Time period: We would expect to find very few pertinent studies prior to 1998, when recovery was first defined in an international policy document[83]. Thus, the precise time frame for the review is 1998 to December 2016.

Setting: Research settings may include inpatient, outpatient or community-based mental health services.

Exclusion criteria: Non-research studies (e.g. editorials, letters, conference abstracts), as well as unpublished (grey) literature, dissertations, and book or book-length studies, will be excluded, as well as conceptual papers and review articles. Studies on services for addiction populations will also be excluded, as recovery is conceptualized differently in the addictions field. Language restrictions will not apply.

Information sources

Our final search strategy will be developed in consultation with an experienced research librarian on the project and will combine a broad, systematic search of the literature. Electronic search will be conducted on the following databases: PubMed, Ovid Medline, Ovid Embase, Ovid PsycInfo, CINAHL (Current Index to Nursing and Allied Health Literature), the Cochrane Library and Scopus. We will supplement our results by conducting the following secondary searches: (a) Reference lists of all selected full text articles will be scanned for additional relevant studies; (b) Citation-tracking will be performed on included articles; (c) Hand searches of tables of contents for the past 5 years will be conducted in the following key journals:

For peer review only - http://bmjopen.bmj.com/site/about/guidelines.xhtml

Psychiatric Rehabilitation Journal; *Psychiatric Services*; and *Community Mental Health Journal*. Additional journals will be added if warranted; and (d) Known experts in the field will be contacted for comments and advice. We will also stay alert to serendipitous discovery that may increase results.

Pilot Search strategy

Using the Ovid Medline database, a research librarian and co-investigator on the project conducted a pilot search (Appendix 1) which generated 5164 records. For this preliminary scoping phase, the search strategy was designed to focus on 3 main components: *mental health*, *recovery* and *services*. Medical Subject Heading (MeSH) and synonyms (keywords) were combined for each of the components. While keywords will remain consistent throughout the searches, subject headings will be revised to reflect database specific preferences. Search strategies will be further revised as new subject headings and keywords are revealed.

Study records

Data management

Electronic search results will be downloaded into EndNote reference manager software, duplicates will be removed where possible, and the remaining references will be uploaded to the Distiller Systematic Review software for the screening and data extraction stages. Distiller software stores references, manages and monitors the screening and data extraction process with customized forms and automated flowcharts, and provides an audit trail for the review.

Screening and Selection process

For the first selection, two team members working independently will read titles and abstracts of each paper identified in the electronic search and assess them for relevance based

For peer review only - http://bmjopen.bmj.com/site/about/guidelines.xhtml

on the inclusion and exclusion criteria. Second, the team members will read the full text of each selected article in order to confirm its inclusion in the study. Disagreements related to the inclusion of any paper will be discussed and resolved, involving a third team member if necessary. To ensure high inter-rater reliability, training exercises will be conducted prior to initiating the screening process. Team members will meet on a weekly basis to follow up on the screening process and discuss unanticipated problems.

Data items and data extraction process

In order to minimize bias, two research team members will independently extract the data. Sample elements for data extraction appear below in Table 1. The categories on the extraction grid include methodological elements based on the PICO mnemonic (PICO= population, intervention, comparison, outcome[84]) for quantitative studies. For qualitative studies, identification of data elements will correspond to the 4 research questions, and will also be guided by the 5 implementation frameworks[85-89] which, taken together, provide myriad implementation processes at different conceptual levels. For example, the 5 dimensions in the Damschroder Consolidated Framework for Advancing Implementation Science[86] include structural, intervention, individual, and "inner"/"outer" setting characteristics that describe the implementation process at the organizational level. Whereas Powell et al[88] provide 68 implementation strategies within 6 processes (planning, educating, financing, restructuring, quality management and policy) involved in clinical practice. Study limitations and gaps in knowledge will also be recorded[90]. The data extraction form will be pre-tested by the two reviewers and revised as needed. Distiller SR software will be used to manage the data extraction process.

For peer review only - http://bmjopen.bmj.com/site/about/guidelines.xhtml

Table 1: Sample elements for data extraction by research question

Study ID/ Methods:	Meaning of Recovery (Q1)	Recovery Implementation (Q2)	Barriers/ Facilitators (Q3)	Recovery Outcomes (Q4)
I.D. #; location<br>Objective<br>Study design<br>Participants<br>Interventions/ comparisons<br>Data collection<br>Data analysis/ triangulation<br>Quality appraisal	Definitions<br>Conceptualizations	Consolidated framework for advancing implementation science (Damschroder et al[91])<br><br>68 strategies for implementation of clinical innovations (Powell et al[92])	Consolidated framework (Damschroder et al)	Theoretical domains framework (Cane et al[85])<br><br>Behavior change technique taxonomy (Michie et al[87])<br><br>Conclusions<br>Study limitations/ gaps/ contradictions/ further questions

Quality assessment

Systematic reviews will be assessed for quality based on the Assessing Methodological Quality of Systematic Reviews tool (AMSTAR) protocol. The AMSTAR is an 11-item questionnaire that assesses study design, literature searched, and scientific quality of reviews; a rating system is included. For primary research studies, quality assessment will be determined using criteria developed by Kmet (2004)[93]. This tool includes a 14-item checklist for quality criteria in quantitative studies, and a 10-item checklist for qualitative studies. A rating system (yes-2; partial -1; and no-0) is provided, as well as a calculation for summary scores. While some controversy exists on whether qualitative research should be assessed using standard quality criteria[94], we will include a quality appraisal for qualitative, as well as quantitative, studies in order to better assess the strengths and weaknesses of the evidence[95]. The selected studies will be independently assessed for quality by two reviewers. Discrepancies will be solved in consultation with the principal investigator.

Data Synthesis

For peer review only - http://bmjopen.bmj.com/site/about/guidelines.xhtml

No single unifying framework exists for synthesizing quantitative and qualitative evidence for healthcare policy-makers and managers[96]. Our experience with recovery research suggests that much of the pertinent literature for review will be qualitative. Thus, our overall approach will be to convert all the evidence into qualitative form. The quantitative data will be transformed into qualitative form by extracting key concepts and findings within the elements geared to our research questions, as described above. Analytic procedures and synthesis will follow a 3-stage process: (a) organization of studies into logical categories according to their design, and methodology; and coding using NVivo 11 software; (b) within-study analysis, according to the study questions; (c) cross-study synthesis of the data using an adaptation of the stepwise thematic analysis developed by Lucas et al[95], according to the following procedures: (1) two reviewers will independently review data collated under each of the research questions; (2) codes produced by each researcher will be compared, and a consolidated list of themes produced for each research question; (3) themes occurring under each question will be clustered around common dimensions; (4) results of the thematic analysis will be presented to the research team at a consensus meeting.

Specific measures will be taken to enhance the trustworthiness of the data. As suggested by Lucas et al, directly reported participant data (e.g. verbatim quotations, or scores on attitudinal scales), and author interpretations, will be reported separately in order to retain the richness or "thickness" of the data. Detailed descriptions, contextual material, and the quality assessment of each paper will also help readers make judgments about the reliability and validity of the data. Summary tables will include counts of the papers contributing data on each theme[94].

For peer review only - http://bmjopen.bmj.com/site/about/guidelines.xhtml

Dissemination

Knowledge translation will involve collaboration with our international consultants, and knowledge users, who include decision makers and managers, service providers, people with lived experience and families. Four output documents will be developed, including: 1) a critical appraisal of findings from the synthesis on recovery implementation; 2) a compendium of case studies on successful recovery implementation initiatives (sensitive to gender, race, culture); 3) a recovery implementation manual for decision makers and managers; and 4) a toolkit of recovery-enhancing practices, i.e. strategies for individual behavior change targeted at service providers and service users. Each document will be submitted to the entire team for revision and editing.

The results of the synthesis project will be widely disseminated. Knowledge translation activities will include: (a) creation of an Advisory Committee composed of the research team, Knowledge Users and international expert advisors. Quarterly telephone meetings with the Advisory to review emerging findings and provide feedback; (b) ongoing consultation/feedback between knowledge users and the research team during preparation of the 4 project outputs; (c) posting of information and updates on the websites of the organizations of knowledge users on the project, including those for people with lived experience and families; (d) organization of a one-day end-of-project symposium for dissemination of project outputs, including workshops for recording feedback and recommendations; (e) dissemination of project outputs through organizational websites; and through national and international networks (free access); (f) submission of articles to peer-reviewed, open access journals; (g) presentations at national/international conferences;

For peer review only - http://bmjopen.bmj.com/site/about/guidelines.xhtml

Conclusion

The recovery approach emerged through the lived experiences of people with enduring mental health problems as they utilized the formal mental health care system. Recovery-oriented services are viewed as a more person-centered and promising approach for treating mental illness. Until now, there has been little access to knowledge concerning how mainstream mental health services are being transformed to a recovery-orientation, and with what results. Our synthesis will establish the state of knowledge and evidence on implementing recovery, and will make this knowledge available to a wide range of mental health stakeholders through dissemination activities and the publication of concrete practice tools. Results may also support the development of new recovery interventions, on which future outcome research should be considered.

Contributors:

MP is the guarantor. ES, JS and MP drafted the manuscript. All authors contributed to the development of the protocol, selection criteria, and data extraction criteria. MP and JS developed the search strategy. All authors read, provided feedback and approved the final manuscript.

Funding:

This study was funded by the Canadian Institute of Health Research, Grant # KRS 144043.

Competing interests: None

For peer review only - http://bmjopen.bmj.com/site/about/guidelines.xhtml

For peer review only

For peer review only - http://bmjopen.bmj.com/site/about/guidelines.xhtml

REFERENCES

1. Adams N, Daniels A, Compagni A. International pathways to mental health transformation. *International Journal of Mental Health.* 2009;38(1):30-45.
2. Kirby M, Keon WJ. *Out of the shadows at last. Transforming mental health, mental illness and addiction services in Canada.* Ottawa: Sénat du Canada;2006.
3. Slade M, Williams J, Bird V, Leamy M, C L. Recovery grows up. *Journal of Mental Health.* 2012;21(2):99-103.
4. Mental Health Commission of Canada. *Changing directions, changing lives. The mental health strategy for Canada.* Calgary, AB2012.
5. Department of Health. The Action Plan for Mental Health in New Brunswick 2011-18. 2011:19.
6. Government of Manitoba. Rising to the challenge. A strategic plan for the mental health and well-being of Manitobans. 2011.
7. Ministère de la Santé et des Services sociaux (MSSS). *Plan d'action en santé mentale 2005-2010 : La force des liens.* Québec: MSSS;2005.
8. Piat M, Sabetti J. Recovery in Canada: Toward social equality. *International Review of Psychiatry.* 2012;24(1):19-28.
9. Ådnøy Eriksen K, Arman M, Davidson L, Sundfør B, Karlsson B. Challenges in relating to mental health professionals: Perspectives of persons with severe mental illness. *International Journal of Mental Health Nursing.* 2014;23(2):110-117.
10. Deegan PE. Recovery: The lived experience of rehabilitation. *Psychosocial Rehabilitation Journal.* 1988;11(4):11-19.
11. Gravel K, Légaré F, Graham ID. Barriers and facilitators to implementing shared decision-making in clinical practice: a systematic review of health professionals' perceptions. *Implementation Science.* 2006;1:16.
12. Légaré F, Ratte S, Gravel K, Graham ID. Barriers and facilitators to implementing shared decision-making in clinical practice: update of a systematic review of health professionals' perceptions. *Patient Education and Counseling.* 2008;73(3):526-535.
13. Nordt C, Rossler W, Lauber C. Attitudes of mental health professionals toward people with schizophrenia and major depression. *Schizophrenia Bulletin.* 2006;32(4):709-714.
14. Brohan E, Elgie R, Sartorious N, Thornicroft G. Self-stigma, empowerment and perceived discrimination among people with schizophrenia in 14 European countries: The GAMIAN-Europe study. *Schizophrenia Research.* 2010;122:232-238.
15. Corrigan PW, Watson AC, Barr L. The self-stigma of mental illness: Implications for self-esteem and self-efficacy. *Journal of Social and Clinical Psychology.* 2006;25(8):875-884.
16. Shahar G, Trower P, Iqbal Z, Birchwood M, Davidson L, Chadwick P. The person in recovery from acute and severe psychosis: the role of dependency, self-criticism, and efficacy. *American Journal of Orthopsychiatry.* 2004;74(4):480-488.
17. Watson AC, Corrigan P, Larson JE, Sells M. Self-stigma in people with mental illness. *Schizophrenia Bulletin.* 2007;33(6):1312-1318.
18. Davidson L, Chan KK. Common factors: evidence-based practice and recovery. *Psychiatric Services.* 2014;65(5):675-677.
19. Joosten EA, DeFuentes-Merillas L, de Weert GH, Sensky T, van der Staak CP, de Jong CA. Systematic review of the effects of shared decision-making on patient satisfaction, treatment adherence and health status. *Psychotherapy and Psychosomatics.* 2008;77(4):219-226.
20. Klockmo C, Marnetoft SU, Selander J, Nordenmark M. Important components to create personal working alliances with clients in the mental health sector to support the recovery process. *International Journal of Rehabilitation Research.* 2014;37(1):40-47.

For peer review only - http://bmjopen.bmj.com/site/about/guidelines.xhtml

21. Sweeney A, Fahmy S, Nolan F, et al. The relationship between therapeutic alliance and service user satisfaction in mental health inpatient wards and crisis house alternatives: A cross-sectional study. *PloS one.* 2014;9(7).

22. Borg M, Kristiansen K. Recovery-oriented professionals: Helping relationships in mental health services. *Journal of Mental Health.* 2004;13(5):493-505.

23. Crowe TP, Deane FP, Oades LG, Caputi P, Morland KG. Effectiveness of a collaborative recovery training program in Australia in promoting positive views about recovery. *Psychiatric Services.* 2006;57(10):1497-1500.

24. Green CA, Janoff SL, Yarborough BJ, Paulson RI. The recovery group project: development of an intervention led jointly by peer and professional counselors. *PsychiatrIC Services.* 2013;64(12):1211-1217.

25. Atterbury K. Preserving the person: The ethical imperative of recovery-oriented practices. *American Journal of Orthopsychiatry.* 2014;84(2):182-189.

26. Andresen R, Caputi P, Oades L. Stages of recovery instrument: development of a measure of recovery from serious mental illness. *Australian and New Zealand Journal of Psychiatry.* 2006;40(11-12):972-980.

27. Giffort D, Schmook A, Woody C, Vollendorf C, Gervain M. *Recovery Assessment Scale.* Chicago: Illinois Department of Mental Health;1995.

28. Khanam D, McDonald K, Williams Neils C. *Measuring recovery: A toolkit for mental health providers in New York City.* New York: The Bureau of Mental Health, NYC Department of Health and Mental Hygiene;2013.

29. Mueser KT, Gingerich S, Salyers MP, McGuire AB, Reyes RU, Cunningham H. *The Illness Management and Recovery (IMR) Scales (client and clinician versions).* Concord, N.H.: New Hampshire-Dartmouth Psychiatric Research Center;2004.

30. Neil ST, Kilbride M, Pitt L, et al. The questionnaire about the process of recovery (QPR): A measurement tool developed in collaboration with service users. *Psychosis.* 2009;1(2):145-155.

31. MacKeith J, Burns S. *Mental Health Recovery Star.* London: Mental Health Providers Forum and Triangle Consulting;2008.

32. Gordon SE, Ellis PM, Siegert RJ, Walkey FH. Core dimensions of recovery: A psychometric analysis. *Administration and Policy in Mental Health and Mental Health Services Research.* 2014;41(4):535-542.

33. Armstrong NP, Steffen JJ. The recovery promotion fidelity scale: assessing the organizational promotion of recovery. *Community Mental Health Journal.* 2009;45(3):163-170.

34. Bedregal LE, O'Connell M, Davidson L. The recovery knowledge inventory: Assessment of mental health staff knowledge and attitudes about recovery. *Psychiatric Rehabilitation Journal.* 2006;30(2):96-103.

35. Burgess P, Pirkis J, Coombs T, Rosen A. Assessing the value of existing recovery measures for routine use in Australian mental health services. *Australian and New Zealand Journal of Psychiatry.* 2011;45(4):267-280.

36. O'Connell M, Tondora J, Croog G, Evans A, Davidson L. From rhetoric to routine: assessing perceptions of recovery-oriented practices in a state mental health and addiction system. *Psychiatric Rehabilitation Journal.* 2005;28(4):378-386.

37. Onken SJ, Dumont JM, Ridgway P, Dornan DH, Ralph RO. Mental Health Recovery: What Helps and What Hinders? A National Research Project for the Development of Recovery Facilitating System Performance Indicators. Piloting the Recovery Oriented System Indicators (ROSI). 2006; http://www.familymentalhealthrecovery.org/conference/handouts/Workshop%208/Onken-ROSIPilotMeasuresV6.pdf. Accessed November 1, 2007.

For peer review only - http://bmjopen.bmj.com/site/about/guidelines.xhtml

38. Ridgway P, Press A. *Assessing the recovery-orientation of your mental health program. A User's guide for the Recovery-enhancing environment scale (REE).* Kansas: University of Kansas;2004.
39. Leamy M, Clarke E, Le Boutillier C, et al. Implementing a complex intervention to support personal recovery: A qualitative study nested within a cluster randomised controlled trial. *PloS one.* 2014;9(5).
40. Lloyd-Evans B, Mayo-Wilson E, Harrison B, et al. A systematic review and meta-analysis of randomised controlled trials of peer support for people with severe mental illness. *BMC Psychiatry.* 2014;14(1).
41. Russinova Z, Rogers ES, Ellison ML, Lyass A. Recovery-promoting professional competencies: perspectives of mental health consumers, consumer-providers and providers. *Psychiatric Rehabilitation Journal.* 2011;34(3):177-185.
42. National Institute for Mental Health in England. *Support, time and recovery (STR) workers. A competence framework.* London: Department of Health;2008.
43. National Social Inclusion Programme. *Capabilities for inclusive practice.* London: Department of Health;2007.
44. National Institute for Mental Health in England. *The ten essential shared capabilities. A framework for the whole of the mental health workforce.* London: Department of Health;2004. 40339.
45. New Zealand Ministry of Health. *Let's get real: Real skills for people working in mental health and addiction.* Wellington2008.
46. Leamy M, Bird V, Le Boutillier C, Williams J, Slade M. Conceptual framework for personal recovery in mental health: systematic review and narrative synthesis. *Br J Psychiatry.* 2011;199(6):445-452.
47. Williams J, Leamy M, Bird V, et al. Measures of the recovery orientation of mental health services: systematic review. *Soc Psychiatry Psychiatr Epidemiol.* 2012;47(11):1827-1835.
48. Slade M, Leamy M, Bacon F, et al. International differences in understanding recovery: Systematic review. *Epidemiology and psychiatric sciences.* 2012;21(4):353-364.
49. Bird V, Leamy M, Tew J, Le Boutillier C, Williams J, Slade M. Fit for purpose? Validation of a conceptual framework for personal recovery with current mental health consumers. *Australian and New Zealand Journal of Psychiatry.* 2014;48(7):644-653.
50. Rappaport J. Terms of empowerment/exemplars of prevention: Toward a theory for community psychology. *American Journal of Community Psychology.* 1987;15(2):121-148.
51. Zimmerman MA. Empowerment theory: Psychological, organizational and community levels of analysis. In: Rappaport J, Seidman E, eds. *Handbook of Community Psychology*. New York: Kluwer Academic/Plenum Publishers; 2000:43-63.
52. Rapp CA, Goscha RJ. *The Strengths Model: Case management with people with psychiatric disabilities.* Oxford, New Yord: Oxford University Press; 2006.
53. Burchardt T. Capabilities and disability: The capabilities framework and the social model of disability. *Disability and Society.* 2004;19(7):735-751.
54. Davidson L, Ridgway P, Wieland M, O'Connell M. A capabilities approach to mental health transformation: A conceptual framework for the recovery era. *Canadian journal of community mental health.* 2009;28(2):35-46.
55. Hopper K. Rethinking social recovery in schizophrenia: What a capabilities approach might offer. *Social Science and Medicine.* 2007;65(5):868-879.
56. Moran GS, Nemec PB. Walking on the sunny side: what positive psychology can contribute to psychiatric rehabilitation concepts and practice. *Psychiatric Rehabilitation Journal.* 2013;36(3):202-208.

For peer review only - http://bmjopen.bmj.com/site/about/guidelines.xhtml

57. Resnick SG, Rosenheck RA. Recovery and positive psychology: Parallel themes and potential synergies. *Psychiatric Services.* 2006;57(1):120-122.
58. Slade M. Mental illness and well-being: The central importance of positive psychology and recovery approaches. *BMC Health Services Research.* 2010;10.
59. Borg M, Karlsson B, Tondora J, Davidson L. Implementing person-centered care in psychiatric rehabilitation: what does this involve? *The Israel Journal of Psychiatry and Related Sciences.* 2009;46(2):84-93.
60. Rudnick A, Roe D. *Serious Mental Illness: Person-Centered Approaches.* London (GB): Radcliffe Publishing Ltd; 2011; 2014 Book Review.
61. Boyle D, Harris M. *The challenge of co-production. How equal partnerships between professionals and the public are crucial to improving public services.* London: NESTA;2009.
62. Denham-Vaughan S, Clark M. Care clusters in mental health and co-production of care-Towards a more lay friendly set of cluster descriptions. *Mental Health and Social Inclusion.* 2012;16(2):79-83.
63. Le Boutillier C, Leamy M, Bird VJ, Davidson L, Williams J, Slade M. What does recovery mean in practice? A qualitative analysis of international recovery-oriented practice guidance. *Psychiatric Services.* 2011;62(12):1470-1476.
64. Sowers W. Transforming systems of care: The American Association of Community Psychiatrists guidelines for recovery oriented services. *Community Mental Health Journal.* 2005;41(6):757-774.
65. Shepherd G, Boardman J, Slade M. *Making recovery a reality.* Sainsbury Centre for Mental Health;2008.
66. Shepherd G, Boardman J, Slade M. *Implementing recovery: A new framework for organisational change.* Sainsbury Centre for Mental Health;2009.
67. Australian Health Ministers' Advisory Council. *A national framework for recovery-oriented mental health services. Guide for practitioners and providers.* Canberra: Department of Health and Ageing;2013.
68. Newnham EA, Page AC. Bridging the gap between best evidence and best practice in mental health. *Clin Psychol Rev.* 2010;30(1):127-142.
69. Anthony WA, Mizock L. Evidence-Based Processes in an Era of Recovery: Implications for Rehabilitation Counseling and Research. *Rehabilitation Counseling Bulletin.* 2014;57(4):219-227.
70. Brown TT, Mahoney CB, Adams N, Felton M, Pareja C. What predicts recovery orientation in county departments of mental health? A pilot study. *Adm Policy Ment Health.* 2010;37(5):388-398.
71. McLoughlin KA, Fitzpatrick JJ. Self-reports of recovery-oriented practices of mental health nurses in state mental health institutes: development of a measure. *Issues in Mental Health Nursing.* 2008;29(10):1051-1065.
72. Osborn LA, Stein CH. Mental Health Care Providers' Views of Their Work with Consumers and Their Reports of Recovery-Orientation, Job Satisfaction, and Personal Growth. *Community Ment Health J.* 2015.
73. Leamy M, Clarke E, Le Boutillier C, et al. A national survey of recovery practice in community mental health teams. *British Journal of Psychiatry.* 2016;209(4):340-346.
74. Chester P, Ehrlich C, Warburton L, Baker D, Kendall E, Crompton D. "What is the work of Recovery Oriented Practice? A systematic literature review". *International Journal of Mental Health Nursing.* 2016;25(4):270-285.
75. Le Boutillier C, Chevalier A, Lawrence V, et al. Staff understanding of recovery-orientated mental health practice: A systematic review and narrative synthesis. *Implementation Science.* 2015.
76. Mental disorders - Fact Sheet No. 396 [press release]. 2014.

For peer review only - http://bmjopen.bmj.com/site/about/guidelines.xhtml

77. Knapp M, Andrew A, McDaid D, et al. *Investing in recovery. Making the business case for effective interventions for people with schizophrenia and psychosis.* London, UK: Center for Mental Health;2014.
78. Latimer EA, Bond GR, Drake RE. Economic approaches to improving access to evidence-based and recovery-oriented services for people with severe mental illness. *Canadian Journal of Psychiatry.* 2011;56(9):523-529.
79. Shamseer L, Moher D, Clarke M, et al. Preferred reporting items for systematic review and meta-analysis protocols (prisma-p) 2015: Elaboration and explanation. *BMJ (Online).* 2015;349.
80. Heyvaert M, Maes B, Onghena P. Mixed methods research synthesis: Definition, framework, and potential. *Quality and Quantity.* 2013;47(2):659-676.
81. Pluye P, Nha Hong Q. Combining the power of stories and the power of numbers: Mixed methods research and mixed studies reviews. *Annual Review of Public Health.* Vol 35: Annual Reviews Inc.; 2014:29-45.
82. Pluye P, Gagnon MP, Griffiths F, Johnson-Lafleur J. A scoring system for appraising mixed methods research, and concomitantly appraising qualitative, quantitative and mixed methods primary studies in Mixed Studies Reviews. *International Journal of Nursing Studies.* 2009;46(4):529-546.
83. New Zealand Mental Health Commission. *Blueprint for mental health services in New Zealand. How things need to be.* Wellington: Mental Health Commission;1998.
84. Richardson WS, Wilson MC, Nishikawa J, Hayward RS. The well-built clinical question: a key to evidence-based decisions. *ACP J Club.* 1995;123(3):A12-13.
85. Cane J, O'Connor D, Michie S. Validation of the theoretical domains framework for use in behaviour change and implementation research. *Implementation Science.* 2012;7(1):37.
86. Damschroder LJ, Aron DC, Keith RE, Kirsh SR, Alexander JA, Lowery JC. Fostering implementation of health services research findings into practice: a consolidated framework for advancing implementation science. *Implementation Science.* 2009;4:50.
87. Michie S, Richardson M, Johnston M, et al. The behavior change technique taxonomy (v1) of 93 hierarchically clustered techniques: Building an international consensus for the reporting of behavior change interventions. *Annals of Behavioral Medicine.* 2013;46(1):81-95.
88. Powell BJ, McMillen JC, Proctor EK, et al. A compilation of strategies for implementing clinical innovations in health and mental health. *Medical Care Research and Review.* 2012;69(2):123-157.
89. Proctor E, Silmere H, Raghavan R, et al. Outcomes for implementation research: conceptual distinctions, measurement challenges, and research agenda. *Adm Policy Ment Health.* 2011;38(2):65-76.
90. Munn Z, Tufanaru C, Aromataris E. Data extraction and synthesis. *American Journal of Nursing.* 2014;114(7):49-54.
91. Damschroder LJ, Aron DC, Keith RE, Kirsh SR, Alexander JA, Lowery JC. Fostering implementation of health services research findings into practice: a consolidated framework for advancing implementation science. *Implement Sci.* 2009;4:50.
92. Powell BJ, McMillen JC, Proctor EK, et al. A compilation of strategies for implementing clinical innovations in health and mental health. *Med Care Res Rev.* 2012;69(2):123-157.
93. Kmet LM, Lee RC, Cook LS. *Standard quality assessment criteria for evaluating primary research papers from a variety of fields.* Alberta Heritage Foundation for Medical Research (AHFMR);2004. HTA Initiative #13.
94. Dixon-Woods M, Agarwal S, Jones D, Young B, Sutton A. Synthesising qualitative and quantitative evidence: A review of possible methods. *Journal of Health Services Research and Policy.* 2005;10(1):45-53.

For peer review only - http://bmjopen.bmj.com/site/about/guidelines.xhtml

95. Lucas PJ, Baird J, Arai L, Law C, Roberts HM. Worked examples of alternative methods for the synthesis of qualitative and quantitative research in systematic reviews. *BMC medical research methodology.* 2007;7.
96. Pope C, Mays N, Popay J. How can we synthesize qualitative and quantitative evidence for healthcare policy-makers and managers? *Healthcare Management Forum.* 2006;19(1):27-31.

For peer review only

For peer review only - http://bmjopen.bmj.com/site/about/guidelines.xhtml

Appendix 1

Pilot search strategy for Ovid MEDLINE(R) In-Process & Other Non-Indexed Citations, Ovid MEDLINE(R) Daily, Ovid MEDLINE(R) and Ovid OLDMEDLINE(R) 1946 to Present

#	Searches
1	recovery oriented.ti,ab,kw.
2	exp Mental Health Services/
3	recover*.ti,ab,kw.
4	2 and 3
5	Mental Health/
6	mental disorders/ or adjustment disorders/ or exp anxiety disorders/ or exp delirium, dementia, amnestic, cognitive disorders/ or exp dissociative disorders/ or exp mood disorders/ or exp neurotic disorders/ or exp personality disorders/ or exp "schizophrenia and disorders with psychotic features"/
7	((Mental or psychiatric or psychologic*) adj2 (health or illness* or disorder* or disease* or problem* or issue or issues or well being)).ti,ab,kw.
8	((adjustment or anxiety or obsessive or complusive or panic or phobic or stress or dissociative or mood or depressive or affective or personality or psychotic or paranoid) adj2 (disorder* or disease*)).ti,ab,kw.
9	(agoraphobi* or astheni* or phobia* or delirium or dementia* or depression or schizophreni* or schizoid).ti,ab,kw.
10	5 or 6 or 7 or 8 or 9
11	exp health services/ or service*.ti,ab,kw.
12	10 and 11
13	exp "Outcome and Process Assessment (Health Care)"/
14	exp delivery of health care/
15	Resource Allocation/
16	exp "Organization and Administration"/
17	exp "Quality of Health Care"/
18	exp professional competence/
19	exp Inservice Training/

For peer review only - http://bmjopen.bmj.com/site/about/guidelines.xhtml

20 exp patient care management/

21 ((service* or organization* or organisation* or unit or units or department* or program* or clinic or clinics or ward or wards or staff or workforce or setting) adj4 (leader* or innovate* or objective* or change* or transform* or structure* or opportunit* or strength* or model* or priorit* or policy or policies or procedure* or ration* or allocat* or reform* or commit* or needs or improve* or assess* or responsive* or evaluat* or plan*)).ti,ab,kw.

22 ((treatment or therapy) adj2 planning).ti,ab,kw.

23 13 or 14 or 15 or 16 or 17 or 18 or 19 or 20 or 21 or 22

24 exp Patient-Centered Care/

25 exp Self Care/ or exp Self Concept/

26 ((self or personal) adj2 (identity or concept or assess* or manage* or disclos* or efficacy or evaluat*)).ti,ab,kw.

27 ((patient or client or consumer*) adj4 (centered or centred or focus* or engage* or involve*)).ti,ab,kw.

28 ((shared or joint) adj2 decision*).ti,ab,kw.

29 exp Interpersonal Relations/

30 "Quality of Life"/ or quality of life.mp.

31 Social Support/ or Social Adjustment/

32 (social adj2 (support or integration or inclusion or adjustment)).ti,ab,kw.

33 (connected* or hope* or optimis* or trust or identity or meaning or empower* or spiritual* or giving back or stigma* or values or goal or goals or belonging or purpose or choice or citizenship or discriminat* or inequalit* or valued or roles).ti,ab,kw.

34 (partnership* or collaborat* or interpersonal or multidisciplinary or cooperat* or co-operat* or team* or group or joint).ti,ab,kw.

35 24 or 25 or 26 or 27 or 28 or 29 or 30 or 31 or 32 or 33 or 34

36 exp Data Collection/

37 exp Psychological Tests/

38 (measur* or test* or scale* or metric* or psychometric* oe questionnaire* or instrument* or data).ti,ab,kw.

39 36 or 37 or 38

40 23 or 39

41 12 and 40

For peer review only - http://bmjopen.bmj.com/site/about/guidelines.xhtml

42 41 and recover*.mp.

43 1 or 4 or 42

For peer review only

For peer review only - http://bmjopen.bmj.com/site/about/guidelines.xhtml

BMJ Open

Protocol for a mixed studies systematic review on the implementation of the recovery approach in adult mental health services

Journal:	*BMJ Open*
Manuscript ID	bmjopen-2017-017080.R1
Article Type:	Protocol
Date Submitted by the Author:	06-Jul-2017
Complete List of Authors:	Piat, Myra; McGill University, Psychiatry; Douglas Mental Health University Institute,<br>Sofouli, Eleni; Douglas Mental Health University Institute<br>Sabetti, Judith; McGill University, School of Social Work<br>Lambrou, Angella; McGill University<br>Chodos, Howard; Mental Health Commission of Canada - Ottawa<br>Briand, Catherine; Inst Univ Sante Mentale Montreal,<br>Vachon, Brigitte ; Universite de Montreal Faculte de medecine<br>Curran, Janet; Dalhousie University - Faculty of Health Professions
**Primary Subject Heading**:	Mental health
Secondary Subject Heading:	Evidence based practice, Health services research
Keywords:	MENTAL HEALTH, Adult psychiatry < PSYCHIATRY, Recovery, HEALTH SERVICES

SCHOLARONE™
Manuscripts

For peer review only

Protocol for a mixed studies systematic review on the implementation of the recovery approach in adult mental health services

Authors:

Myra Piat[1,2,3] ,PhD,
Eleni Sofouli[3] ,MSW,
Judith Sabetti[2,3] ,PhD,
Angella Lambrou[4] ,MLS
Howard Chodos, PhD
Catherine Briand [5], PhD,
Brigitte Vachon [5], PhD,
Janet Curran[6], PhD,

Author Affiliations:

[1]Department of Psychiatry, McGill University, Montreal, QC, Canada
[2]School of Social Work, McGill University, Montreal, QC, Canada
[3]Douglas Mental Health University Institute, Research Center, Montreal, QC, Canada
[4]Schulich Library of Physical Sciences, Life Sciences and Engineering, McGill University (retired)
[5] Faculty of Medicine, University of Montreal, Montreal, QC, Canada
[6] School of Nursing, Faculty of Health Professions, Dalhousie University

Email:
myra.piat@douglas.mcgill.ca
eleni.sofouli@douglas.mcgill.ca
judith.sabetti@douglas.mcgill.ca
angella.lambrou@mcgill.ca
catharine.briand@umontreal.ca
brigitte.vachon@umontreal.ca
hchodos@rogers.com
janet.curran@iwk.nshealth.ca

Corresponding author: Myra Piat, PhD myra.piat@douglas.mcgill.ca

Word count: 3049

For peer review only

For peer review only - http://bmjopen.bmj.com/site/about/guidelines.xhtml

ABSTRACT

Introduction:

Recovery is integral to mental health planning in G-8 countries including Canada. A recovery-oriented approach to care aims to promote personal empowerment, illness self-management, and a life beyond services for people with serious mental illness (SMI), while reducing the staggering financial burden associated with mental illness. Although there is a growing body of literature on recovery-oriented services, no synthesis of research on the implementation of recovery into mental health services exists.

Method and analysis:

A mixed studies systematic review will be conducted on recovery-oriented services for adults with SMI. Seven databases including PubMed, Ovid Medline, Ovid Embase, Ovid PsycInfo, CINAHL (Current Index to Nursing and Allied Health Literature), the Cochrane Library and Scopus will be searched for peer-reviewed empirical studies published from 1998 to December 2016. Systematic reviews, and studies using quantitative, qualitative and mixed methodologies will be included. Secondary searches will be conducted in reference lists of all selected full text articles; and citation-tracking will be performed on articles related to those included in the review. Hand searches will also be performed in the tables of contents of three recovery-focused journals for the most recent 5 years. International experts in the field will be contacted for comments and advice. Data extraction will include identification and methodological synthesis of each study; definition of recovery; information on recovery implementation; facilitators and barriers; and study outcomes. A quality assessment will be conducted on each study. The data will be synthesized and a stepwise thematic analysis performed.

Ethics and dissemination:

Ethics approval is not required for this knowledge synthesis. Findings will be disseminated through multiple knowledge translation activities including: a) a one-day symposium; b) presentations in national and international conferences, and to local stakeholders; c) publications in peer-reviewed open access journals, d) posts on the organizational websites of participating knowledge users.

For peer review only

For peer review only - http://bmjopen.bmj.com/site/about/guidelines.xhtml

Strengths and limitations of this study

- Studies included in this knowledge synthesis will have been conducted in inpatient, outpatient and community-based mental health settings, and will cover a broad range of research methodologies. The synthesis will reveal how recovery is understood; challenges involved in implementation; and, overall, to what extent transformation to recovery oriented services and systems is occurring.
- The selection of recovery-oriented studies with an implementation focus is unique, and will allow us to draw on one powerful conceptual models from implementation science that provide theory-informed elements to guide data analysis and synthesis as well as the reporting of results.
- Knowledge from the synthesis will be compiled into comprehensive and usable formats for organizational and government stakeholders, providing practical guidelines for recovery-based service reform and future evaluation.
- Limiting the search to published, peer-reviewed studies, while important for considerations of quality and methodological rigor, may overlook possible research on recovery-oriented services reported elsewhere.

INTRODUCTION

Rationale

Recovery is the focus of national mental health plans in G-8 countries[1-3]including Canada's first mental health strategy, *Changing Directions, Changing Lives*[4], and several provincial strategies[5-8]. The rationale for transformation to recovery-oriented services in mental health is compelling. While traditional mental health services have underlined professional control[9-13], reinforcing patient dependency, self-stigma and hopelessness[14-17], recovery approaches focus on individual empowerment, strong collaborative relationships between mental health service providers and service users, and community integration[18-24]. In promoting a life beyond services, recovery also meets a key ethical obligation to honor the personhood and citizenship of people with mental illness[25].

For peer review only

For peer review only - http://bmjopen.bmj.com/site/about/guidelines.xhtml

Recovery knowledge and evidence have burgeoned over the past two decades. Research exists on personal recovery[26-32], recovery-oriented services[33-40], and provider competencies[41-45]. Conceptual frameworks and standardized measures have been produced[35,46-49]. Other studies have linked the recovery approach to recognized theories, such as empowerment theory[50,51], the strengths model[52], capabilities theory[53-55], positive psychology[56-58], person-centered practice[59,60] and co-production[61,62]. Practice guidelines for recovery-oriented service provision are available[63-69], as well. In terms of the empirical literature, studies on particular agencies and programs have identified potential determinants of recovery orientation in services: for example a flexible and innovative organizational culture, results-oriented leadership and larger budgets[70] were found to be associated with recovery-oriented services, as were provider socio-professional characteristics such as greater age, higher educational levels and more professional experience[71,72]. Another recent study found that increasing the recovery orientation of teamwork on mental health teams[73] was associated with provider and consumer perceptions that services were recovery-oriented.

While two recent systematic reviews have been conducted on the recovery-oriented practices of mental health service providers[74,75], no known review has been published, to date, on the implementation of the recovery approaches into mental health services. Our review synthesizes research on the nature of recovery-oriented services, implementation challenges and overall system transformation. Work on the project initiated in August 2016, and should come to completion in spring 2018.

This project is important and timely, as mental illness affects millions of people worldwide. According to recent World Health Organization statistics[76], 350 million people are

For peer review only - http://bmjopen.bmj.com/site/about/guidelines.xhtml

impacted by depression; 60 million people by bipolar disorder, and 21 million affected by schizophrenia. A recovery-oriented approach to healthcare is expected to reduce dependency and reduce the cost of mental healthcare, which in Canada has risen to more than $50 billion per year[4,77,78]. The project responds to a critical knowledge gap identified by knowledge users across Canada, who are responsible for implementing provincial level policy as well as shifting mental health organizations and services to a recovery orientation.

Objectives

The overall goal of this review is to systematically search, assess, and synthesize, implementation studies on mental health recovery from the international mental health literature in order to inform, and facilitate, the transformation of Canadian mental health systems and adult services to a recovery orientation. The following six research questions, guided by the Consolidated Framework for Advancing Implementation Science (CFIR)[79] will be applied to each selected study: (1) How was recovery defined in this study?; (2) How was the recovery approach implemented in this study (Intervention)? (3) What elements from the external environment (Outer setting), or internal environment (Inner setting), influenced implementation in this study?; (4) What were the characteristics of participants in this study? (Characteristics of individuals); (5) What processes were involved in effecting the implementation?; and 6) What was the extent and effectiveness of implementation in this study?

METHODS

Eligibility criteria

For peer review only - http://bmjopen.bmj.com/site/about/guidelines.xhtml

The design and methodology for the present review are reported following the Preferred Reporting Items for Systematic review and Meta-Analysis Protocols (PRISMA-P) guidelines[80] (Appendix 1).

Population: The review concerns studies of services for adults (18 yrs. +) with a primary diagnosis of schizophrenia, bipolar disorder or major depression, following the DSM-V classification for mental disorders.

Intervention: Studies will be included if they describe and evaluate the implementation of any intervention based on recovery principles that aims at transforming the orientation of mental health services, or organizations to a recovery approach.

Comparators: Studies will be eligible for inclusion whether or not they include comparison groups.

Outcomes: Selected studies should report outcomes related to the transformation of a mental health service, or organization, to a recovery-orientation. Outcomes might include change in organizational culture; more integrated service networks and partnerships; increased knowledge, skills and/or attitudinal change among mental health providers; more use of evidence based recovery-oriented best practices; greater consumer/provider collaboration, consumer self-management, and evaluation.

Study design: This will be a mixed studies review (MSR)[81,82]. The MSR integrates qualitative, quantitative and mixed methods studies, providing a rich, detailed understanding of complex health interventions and programs[83]. Studies representing a full range of methodologies will be included: systematic reviews and meta-analyses, randomized control trials (RCTs) and clinical trials, observational, mixed method and qualitative studies.

For peer review only - http://bmjopen.bmj.com/site/about/guidelines.xhtml

Time period: We would expect to find very few pertinent studies prior to 1998, when recovery was first defined in an international policy document[84]. Thus, the precise time frame for the review is 1998 to December 2016.

Setting: Research settings may include inpatient, outpatient or community-based mental health services.

Exclusion criteria: Non-research studies (e.g. editorials, letters, conference abstracts), as well as unpublished (grey) literature, dissertations, and book or book-length studies, will be excluded, as well as conceptual papers and review articles. Studies on services for addiction populations will also be excluded, as recovery is conceptualized differently in the addictions field. Language restrictions will not apply.

Information sources

Our final search strategy will be developed in consultation with an experienced research librarian on the project and will combine a broad, systematic search of the literature. Electronic search will be conducted on the following databases: PubMed, Ovid Medline, Ovid Embase, Ovid PsycInfo, CINAHL (Current Index to Nursing and Allied Health Literature), the Cochrane Library and Scopus. We will supplement our results by conducting the following secondary searches: (a) Reference lists of all selected full text articles will be scanned for additional relevant studies; (b) Citation-tracking will be performed on included articles; (c) Hand searches of tables of contents for the past 5 years will be conducted in the following key journals: *Psychiatric Rehabilitation Journal*; *Psychiatric Services*; and *Community Mental Health Journal*. Additional journals will be added if warranted; and (d) Known experts in the field will be

For peer review only - http://bmjopen.bmj.com/site/about/guidelines.xhtml

For peer review only

contacted for comments and advice. We will also stay alert to serendipitous discovery that may increase results.

Pilot Search strategy

Using the Ovid Medline database, a research librarian and co-investigator on the project conducted a pilot search (Appendix 2) which generated 5164 records. For this preliminary scoping phase, the search strategy was designed to focus on 3 main components: *mental health*, *recovery* and *services*. Medical Subject Heading (MeSH) and synonyms (keywords) were combined for each of the components. Terms related to recovery were chosen to reflect the consumer-survivor understanding of recovery[48,85,86] While keywords will remain consistent throughout the searches, subject headings will be revised to reflect database specific preferences. Search strategies will be further revised as new subject headings and keywords are revealed.

STUDY RECORDS

Data management

Electronic search results will be downloaded into EndNote reference manager software, duplicates will be removed where possible, and the remaining references will be uploaded to the Distiller Systematic Review software for the screening and data extraction stages. Distiller software stores references, manages and monitors the screening and data extraction process with customized forms and automated flowcharts, and provides an audit trail for the review.

Screening and Selection process

For the first selection, two team members working independently will read titles and abstracts of each paper identified in the electronic search and assess them for relevance based

For peer review only - http://bmjopen.bmj.com/site/about/guidelines.xhtml

on the inclusion and exclusion criteria. Second, the team members will read the full text of each selected article in order to confirm its inclusion in the study. Disagreements related to the inclusion of any paper will be discussed and resolved, involving a third team member if necessary. To ensure high inter-rater reliability, training exercises will be conducted prior to initiating the screening process. Team members will meet on a weekly basis to follow up on the screening process and discuss unanticipated problems.

Data items and data extraction process

In order to minimize bias, two research team members will independently extract the data. Sample elements for data extraction appear below in Table 1. The categories on the extraction grid include methodological elements based on the PICO mnemonic (PICO= population, intervention, comparison, outcome[87]). As well, elements corresponding to the 6 research questions will be extracted and organized using the CFIR[79], a multilevel five-dimension determinant framework[88] that constitutes a highly useful tool for identifying barriers and facilitators influencing implementation outcomes. Study limitations and gaps in knowledge will also be recorded[93]. The data extraction form will be pre-tested by the two reviewers and revised as needed. Distiller SR software will be used to manage the data extraction process.

Table 1: Sample elements for data extraction

For peer review only - http://bmjopen.bmj.com/site/about/guidelines.xhtml

Study ID/ Methods	Recovery Definition & Intervention	Characteristics of the Setting		Characteristics of Individuals	Process	Implementation Outcomes
		Outer	Inner			
I.D. #; location<br>Objective<br>Study design<br>Participants<br>Interventions/ comparisons<br>Data collection<br>Data analysis/ triangulation<br>Quality appraisal	-Recovery definition/ conceptualization<br><br>-Intervention Characteristics<br>source<br>adaptability<br>trialability<br>complexity<br>design& packaging<br>costs	-Needs & resources<br>-External links<br>-Political pressures<br>-Policy & incentives	-Structure<br>-Dialogue<br>-Culture<br>-Tension for change<br>-Values & Norms<br>-Priorities<br>- Incentives & rewards	-Knowledge & beliefs<br>-Identification with organization<br>-Other personal attributes	-Advance planning<br>-« Buy-in »)<br>opinion leaders<br>implementation leaders<br>champions<br>-Execution<br>-Feedback	-extent of successful implementation of intervention<br><br>-Conclusions<br>-Study limitations/ gaps/<br>-Contradictions/ further questions

Quality assessment

Systematic reviews require that selected studies are assessed for quality[96]. We will the Assessing Methodological Quality of Systematic Reviews tool (AMSTAR) protocol for the assessment of systematic reviews. The AMSTAR is an 11-item questionnaire that assesses study design, literature searched, and scientific quality of reviews; a rating system is included. For primary research studies, quality assessment will be determined using criteria developed by Kmet (2004)[97]. This tool includes a 14-item checklist for quality criteria in quantitative studies, and a 10-item checklist for qualitative studies. A rating system (yes-2; partial -1; and no-0) is provided, as well as a calculation for summary scores. While some controversy exists on whether qualitative research should be assessed using standard quality criteria[98], we will include a quality appraisal for qualitative, as well as quantitative, studies in order to better assess the strengths and weaknesses of the evidence[99]. The Cochrane Collaboration tool will be used for assessing the risk of bias in randomized control trials[100]. The selected studies will be

For peer review only

For peer review only - http://bmjopen.bmj.com/site/about/guidelines.xhtml

independently assessed for quality by two reviewers. Discrepancies will be solved in consultation with the principal investigator.

DATA

Data Synthesis

No single unifying framework exists for synthesizing quantitative and qualitative evidence for healthcare policy-makers and managers[101]. Our experience with recovery research suggests that much of the pertinent literature for review will be qualitative. Thus, our overall approach will be to convert all the evidence into qualitative form. The quantitative data will be transformed into qualitative form by extracting key concepts and findings within the elements geared to our research questions, as described above. Analytic procedures and synthesis will follow a 3-stage process: (a) organization of studies into logical categories according to their design, and methodology; and coding using NVivo 11 software; (b) within-study analysis, according to the study questions; (c) cross-study synthesis of the data using an adaptation of the stepwise thematic analysis developed by Lucas et al[99], according to the following procedures: (1) two reviewers will independently review data collated under each of the research questions; (2) codes produced by each researcher will be compared, and a consolidated list of themes produced for each research question; (3) themes occurring under each question will be clustered around common dimensions; (4) results of the thematic analysis will be presented to the research team at a consensus meeting.

Specific measures will be taken to enhance the trustworthiness of the data. As suggested by Lucas et al, directly reported participant data (e.g. verbatim quotations, or scores on attitudinal scales), and author interpretations, will be reported separately in order to retain

For peer review only - http://bmjopen.bmj.com/site/about/guidelines.xhtml

the richness or "thickness" of the data. Detailed descriptions, contextual material, and the quality assessment of each paper will also help readers make judgments about the reliability and validity of the data. Summary tables will include counts of the papers contributing data on each theme[98].

DISSEMINATION

Knowledge translation will involve collaboration with our international consultants, and knowledge users, who include decision makers and managers, service providers, people with lived experience and families. Four output documents will be developed, including: 1) a critical appraisal of findings from the synthesis on recovery implementation; 2) a compendium of case studies on successful recovery implementation initiatives (sensitive to gender, race, culture); 3) a recovery implementation manual for decision makers and managers; and 4) a toolkit of recovery-enhancing approaches, i.e. strategies for individual behavior change targeted at service providers and service users. Each document will be submitted to the entire team for revision and editing.

The results of the synthesis project will be widely disseminated. Knowledge translation activities will include: (a) creation of an Advisory Committee composed of the research team, Knowledge Users and international expert advisors. Quarterly telephone meetings with the Advisory to review emerging findings and provide feedback; (b) ongoing consultation/feedback between knowledge users and the research team during preparation of the 4 project outputs; (c) posting of information and updates on the websites of the organizations of knowledge users on the project, including those for people with lived experience and families; (d) organization of a one-day end-of-project symposium for dissemination of project outputs, including workshops

For peer review only - http://bmjopen.bmj.com/site/about/guidelines.xhtml

for recording feedback and recommendations; (e) dissemination of project outputs through organizational websites; and through national and international networks (free access); (f) submission of articles to peer-reviewed, open access journals; (g) presentations at national/international conferences;

CONCLUSION

The recovery approach emerged through the lived experiences of people with enduring mental health problems as they utilized the formal mental health care system. Recovery-oriented services are viewed as a more person-centered and promising approach for treating mental illness. Until now, there has been little access to knowledge concerning how mainstream mental health services are being transformed to a recovery-orientation, and with what results. Our synthesis will establish the state of knowledge and evidence on implementing recovery, and will make this knowledge available to a wide range of mental health stakeholders through dissemination activities and the publication of concrete recovery implementation tools. Results may also support the development of new recovery interventions, on which future outcome research should be considered.

Contributors:

Myra Piat (MP) is the guarantor. Myra Piat (MP), Eleni Sofouli (ES) and Judith Sabetti (JS) drafted the manuscript. Myra Piat (MP) Eleni Sofouli (ES), Judith Sabetti (JS), Catherine Briand (CB), Brigitte Vachon (BV) and Janet Curran (JC) contributed to the development of the selection criteria, and data extraction criteria. Angella Lambrou (AL) developed the preliminary search strategy. Howard Chodos (HC) Catherine Briand (CB), Brigitte Vachon (BV) and Janet Curran (JC) provided written feedback on the manuscript and Angella Lambrou (AL) Howard Chodos (HC) Catherine Briand (CB) Brigitte Vachon (BV) and Janet Curran (JC) approved the final manuscript.

For peer review only - http://bmjopen.bmj.com/site/about/guidelines.xhtml

Funding:

This study was funded by the Canadian Institute of Health Research, grant number KRS 144043.

Competing interests: None

REFERENCES

1. Adams N, Daniels A, Compagni A. International pathways to mental health transformation. *International Journal of Mental Health.* 2009;38(1):30-45.
2. Kirby M, Keon WJ. *Out of the shadows at last. Transforming mental health, mental illness and addiction services in Canada.* Ottawa: Sénat du Canada;2006.
3. Slade M, Williams J, Bird V, Leamy M, C L. Recovery grows up. *Journal of Mental Health.* 2012;21(2):99-103.
4. Mental Health Commission of Canada. *Changing directions, changing lives. The mental health strategy for Canada.* Calgary, AB2012.
5. Department of Health. The Action Plan for Mental Health in New Brunswick 2011-18. 2011:19.
6. Government of Manitoba. Rising to the challenge. A strategic plan for the mental health and well-being of Manitobans. 2011.
7. Ministère de la Santé et des Services sociaux (MSSS). *Plan d'action en santé mentale 2005-2010 : La force des liens.* Québec: MSSS;2005.
8. Piat M, Sabetti J. Recovery in Canada: Toward social equality. *International Review of Psychiatry.* 2012;24(1):19-28.
9. Ådnøy Eriksen K, Arman M, Davidson L, Sundfør B, Karlsson B. Challenges in relating to mental health professionals: Perspectives of persons with severe mental illness. *International Journal of Mental Health Nursing.* 2014;23(2):110-117.
10. Deegan PE. Recovery: The lived experience of rehabilitation. *Psychosocial Rehabilitation Journal.* 1988;11(4):11-19.
11. Gravel K, Légaré F, Graham ID. Barriers and facilitators to implementing shared decision-making in clinical practice: a systematic review of health professionals' perceptions. *Implementation Science.* 2006;1:16.
12. Légaré F, Ratte S, Gravel K, Graham ID. Barriers and facilitators to implementing shared decision-making in clinical practice: update of a systematic review of health professionals' perceptions. *Patient Education and Counseling.* 2008;73(3):526-535.
13. Nordt C, Rossler W, Lauber C. Attitudes of mental health professionals toward people with schizophrenia and major depression. *Schizophrenia Bulletin.* 2006;32(4):709-714.
14. Brohan E, Elgie R, Sartorious N, Thornicroft G. Self-stigma, empowerment and perceived discrimination among people with schizophrenia in 14 European countries: The GAMIAN-Europe study. *Schizophrenia Research.* 2010;122:232-238.
15. Corrigan PW, Watson AC, Barr L. The self-stigma of mental illness: Implications for self-esteem and self-efficacy. *Journal of Social and Clinical Psychology.* 2006;25(8):875-884.
16. Shahar G, Trower P, Iqbal Z, Birchwood M, Davidson L, Chadwick P. The person in recovery from acute and severe psychosis: the role of dependency, self-criticism, and efficacy. *American Journal of Orthopsychiatry.* 2004;74(4):480-488.
17. Watson AC, Corrigan P, Larson JE, Sells M. Self-stigma in people with mental illness. *Schizophrenia Bulletin.* 2007;33(6):1312-1318.
18. Davidson L, Chan KK. Common factors: evidence-based practice and recovery. *Psychiatric Services.* 2014;65(5):675-677.

For peer review only - http://bmjopen.bmj.com/site/about/guidelines.xhtml

19. Joosten EA, DeFuentes-Merillas L, de Weert GH, Sensky T, van der Staak CP, de Jong CA. Systematic review of the effects of shared decision-making on patient satisfaction, treatment adherence and health status. *Psychotherapy and Psychosomatics.* 2008;77(4):219-226.
20. Klockmo C, Marnetoft SU, Selander J, Nordenmark M. Important components to create personal working alliances with clients in the mental health sector to support the recovery process. *International Journal of Rehabilitation Research.* 2014;37(1):40-47.
21. Sweeney A, Fahmy S, Nolan F, et al. The relationship between therapeutic alliance and service user satisfaction in mental health inpatient wards and crisis house alternatives: A cross-sectional study. *PloS one.* 2014;9(7).
22. Borg M, Kristiansen K. Recovery-oriented professionals: Helping relationships in mental health services. *Journal of Mental Health.* 2004;13(5):493-505.
23. Crowe TP, Deane FP, Oades LG, Caputi P, Morland KG. Effectiveness of a collaborative recovery training program in Australia in promoting positive views about recovery. *Psychiatric Services.* 2006;57(10):1497-1500.
24. Green CA, Janoff SL, Yarborough BJ, Paulson RI. The recovery group project: development of an intervention led jointly by peer and professional counselors. *PsychiatrIC Services.* 2013;64(12):1211-1217.
25. Atterbury K. Preserving the person: The ethical imperative of recovery-oriented practices. *American Journal of Orthopsychiatry.* 2014;84(2):182-189.
26. Andresen R, Caputi P, Oades L. Stages of recovery instrument: development of a measure of recovery from serious mental illness. *Australian and New Zealand Journal of Psychiatry.* 2006;40(11-12):972-980.
27. Giffort D, Schmook A, Woody C, Vollendorf C, Gervain M. *Recovery Assessment Scale.* Chicago: Illinois Department of Mental Health;1995.
28. Khanam D, McDonald K, Williams Neils C. *Measuring recovery: A toolkit for mental health providers in New York City.* New York: The Bureau of Mental Health, NYC Department of Health and Mental Hygiene;2013.
29. Mueser KT, Gingerich S, Salyers MP, McGuire AB, Reyes RU, Cunningham H. *The Illness Management and Recovery (IMR) Scales (client and clinician versions).* Concord, N.H.: New Hampshire-Dartmouth Psychiatric Research Center;2004.
30. Neil ST, Kilbride M, Pitt L, et al. The questionnaire about the process of recovery (QPR): A measurement tool developed in collaboration with service users. *Psychosis.* 2009;1(2):145-155.
31. MacKeith J, Burns S. *Mental Health Recovery Star.* London: Mental Health Providers Forum and Triangle Consulting;2008.
32. Gordon SE, Ellis PM, Siegert RJ, Walkey FH. Core dimensions of recovery: A psychometric analysis. *Administration and Policy in Mental Health and Mental Health Services Research.* 2014;41(4):535-542.
33. Armstrong NP, Steffen JJ. The recovery promotion fidelity scale: assessing the organizational promotion of recovery. *Community Mental Health Journal.* 2009;45(3):163-170.
34. Bedregal LE, O'Connell M, Davidson L. The recovery knowledge inventory: Assessment of mental health staff knowledge and attitudes about recovery. *Psychiatric Rehabilitation Journal.* 2006;30(2):96-103.
35. Burgess P, Pirkis J, Coombs T, Rosen A. Assessing the value of existing recovery measures for routine use in Australian mental health services. *Australian and New Zealand Journal of Psychiatry.* 2011;45(4):267-280.
36. O'Connell M, Tondora J, Croog G, Evans A, Davidson L. From rhetoric to routine: assessing perceptions of recovery-oriented practices in a state mental health and addiction system. *Psychiatric Rehabilitation Journal.* 2005;28(4):378-386.

For peer review only

For peer review only - http://bmjopen.bmj.com/site/about/guidelines.xhtml

37. Onken SJ, Dumont JM, Ridgway P, Dornan DH, Ralph RO. Mental Health Recovery: What Helps and What Hinders? A National Research Project for the Development of Recovery Facilitating System Performance Indicators. Piloting the Recovery Oriented System Indicators (ROSI). 2006; http://www.familymentalhealthrecovery.org/conference/handouts/Workshop%208/Onken-ROSIPilotMeasuresV6.pdf. Accessed November 1, 2007.

38. Ridgway P, Press A. *Assessing the recovery-orientation of your mental health program. A User's guide for the Recovery-enhancing environment scale (REE).* Kansas: University of Kansas;2004.

39. Leamy M, Clarke E, Le Boutillier C, et al. Implementing a complex intervention to support personal recovery: A qualitative study nested within a cluster randomised controlled trial. *PloS one.* 2014;9(5).

40. Lloyd-Evans B, Mayo-Wilson E, Harrison B, et al. A systematic review and meta-analysis of randomised controlled trials of peer support for people with severe mental illness. *BMC Psychiatry.* 2014;14(1).

41. Russinova Z, Rogers ES, Ellison ML, Lyass A. Recovery-promoting professional competencies: perspectives of mental health consumers, consumer-providers and providers. *Psychiatric Rehabilitation Journal.* 2011;34(3):177-185.

42. National Institute for Mental Health in England. *Support, time and recovery (STR) workers. A competence framework.* London: Department of Health;2008.

43. National Social Inclusion Programme. *Capabilities for inclusive practice.* London: Department of Health;2007.

44. National Institute for Mental Health in England. *The ten essential shared capabilities. A framework for the whole of the mental health workforce.* London: Department of Health;2004. 40339.

45. New Zealand Ministry of Health. *Let's get real: Real skills for people working in mental health and addiction.* Wellington2008.

46. Leamy M, Bird V, Le Boutillier C, Williams J, Slade M. Conceptual framework for personal recovery in mental health: systematic review and narrative synthesis. *Br J Psychiatry.* 2011;199(6):445-452.

47. Williams J, Leamy M, Bird V, et al. Measures of the recovery orientation of mental health services: systematic review. *Soc Psychiatry Psychiatr Epidemiol.* 2012;47(11):1827-1835.

48. Slade M, Leamy M, Bacon F, et al. International differences in understanding recovery: Systematic review. *Epidemiology and psychiatric sciences.* 2012;21(4):353-364.

49. Bird V, Leamy M, Tew J, Le Boutillier C, Williams J, Slade M. Fit for purpose? Validation of a conceptual framework for personal recovery with current mental health consumers. *Australian and New Zealand Journal of Psychiatry.* 2014;48(7):644-653.

50. Rappaport J. Terms of empowerment/exemplars of prevention: Toward a theory for community psychology. *American Journal of Community Psychology.* 1987;15(2):121-148.

51. Zimmerman MA. Empowerment theory: Psychological, organizational and community levels of analysis. In: Rappaport J, Seidman E, eds. *Handbook of Community Psychology*. New York: Kluwer Academic/Plenum Publishers; 2000:43-63.

52. Rapp CA, Goscha RJ. *The Strengths Model: Case management with people with psychiatric disabilities.* Oxford, New Yord: Oxford University Press; 2006.

53. Burchardt T. Capabilities and disability: The capabilities framework and the social model of disability. *Disability and Society.* 2004;19(7):735-751.

54. Davidson L, Ridgway P, Wieland M, O'Connell M. A capabilities approach to mental health transformation: A conceptual framework for the recovery era. *Canadian journal of community mental health.* 2009;28(2):35-46.

For peer review only

For peer review only - http://bmjopen.bmj.com/site/about/guidelines.xhtml

55. Hopper K. Rethinking social recovery in schizophrenia: What a capabilities approach might offer. *Social Science and Medicine.* 2007;65(5):868-879.
56. Moran GS, Nemec PB. Walking on the sunny side: what positive psychology can contribute to psychiatric rehabilitation concepts and practice. *Psychiatric Rehabilitation Journal.* 2013;36(3):202-208.
57. Resnick SG, Rosenheck RA. Recovery and positive psychology: Parallel themes and potential synergies. *Psychiatric Services.* 2006;57(1):120-122.
58. Slade M. Mental illness and well-being: The central importance of positive psychology and recovery approaches. *BMC Health Services Research.* 2010;10.
59. Borg M, Karlsson B, Tondora J, Davidson L. Implementing person-centered care in psychiatric rehabilitation: what does this involve? *The Israel Journal of Psychiatry and Related Sciences.* 2009;46(2):84-93.
60. Rudnick A, Roe D. *Serious Mental Illness: Person-Centered Approaches.* London (GB): Radcliffe Publishing Ltd; 2011; 2014 Book Review.
61. Boyle D, Harris M. *The challenge of co-production. How equal partnerships between professionals and the public are crucial to improving public services.* London: NESTA;2009.
62. Denham-Vaughan S, Clark M. Care clusters in mental health and co-production of care-Towards a more lay friendly set of cluster descriptions. *Mental Health and Social Inclusion.* 2012;16(2):79-83.
63. Le Boutillier C, Leamy M, Bird VJ, Davidson L, Williams J, Slade M. What does recovery mean in practice? A qualitative analysis of international recovery-oriented practice guidance. *Psychiatric Services.* 2011;62(12):1470-1476.
64. Sowers W. Transforming systems of care: The American Association of Community Psychiatrists guidelines for recovery oriented services. *Community Mental Health Journal.* 2005;41(6):757-774.
65. Shepherd G, Boardman J, Slade M. *Making recovery a reality.* Sainsbury Centre for Mental Health;2008.
66. Shepherd G, Boardman J, Slade M. *Implementing recovery: A new framework for organisational change.* Sainsbury Centre for Mental Health;2009.
67. Australian Health Ministers' Advisory Council. *A national framework for recovery-oriented mental health services. Guide for practitioners and providers.* Canberra: Department of Health and Ageing;2013.
68. Newnham EA, Page AC. Bridging the gap between best evidence and best practice in mental health. *Clin Psychol Rev.* 2010;30(1):127-142.
69. Anthony WA, Mizock L. Evidence-Based Processes in an Era of Recovery: Implications for Rehabilitation Counseling and Research. *Rehabilitation Counseling Bulletin.* 2014;57(4):219-227.
70. Brown TT, Mahoney CB, Adams N, Felton M, Pareja C. What predicts recovery orientation in county departments of mental health? A pilot study. *Adm Policy Ment Health.* 2010;37(5):388-398.
71. McLoughlin KA, Fitzpatrick JJ. Self-reports of recovery-oriented practices of mental health nurses in state mental health institutes: development of a measure. *Issues in Mental Health Nursing.* 2008;29(10):1051-1065.
72. Osborn LA, Stein CH. Mental Health Care Providers' Views of Their Work with Consumers and Their Reports of Recovery-Orientation, Job Satisfaction, and Personal Growth. *Community Ment Health J.* 2015.
73. Leamy M, Clarke E, Le Boutillier C, et al. A national survey of recovery practice in community mental health teams. *British Journal of Psychiatry.* 2016;209(4):340-346.

For peer review only - http://bmjopen.bmj.com/site/about/guidelines.xhtml

74. Chester P, Ehrlich C, Warburton L, Baker D, Kendall E, Crompton D. “What is the work of Recovery Oriented Practice? A systematic literature review”. *International Journal of Mental Health Nursing.* 2016;25(4):270-285.

75. Le Boutillier C, Chevalier A, Lawrence V, et al. Staff understanding of recovery-orientated mental health practice: A systematic review and narrative synthesis. *Implementation Science.* 2015.

76. Mental disorders - Fact Sheet No. 396 [press release]. 2014.

77. Knapp M, Andrew A, McDaid D, et al. *Investing in recovery. Making the business case for effective interventions for people with schizophrenia and psychosis.* London, UK: Center for Mental Health;2014.

78. Latimer EA, Bond GR, Drake RE. Economic approaches to improving access to evidence-based and recovery-oriented services for people with severe mental illness. *Canadian Journal of Psychiatry.* 2011;56(9):523-529.

79. Damschroder LJ, Aron DC, Keith RE, Kirsh SR, Alexander JA, Lowery JC. Fostering implementation of health services research findings into practice: a consolidated framework for advancing implementation science. *Implementation Science.* 2009;4:50.

80. Shamseer L, Moher D, Clarke M, et al. Preferred reporting items for systematic review and meta-analysis protocols (prisma-p) 2015: Elaboration and explanation. *BMJ (Online).* 2015;349.

81. Heyvaert M, Maes B, Onghena P. Mixed methods research synthesis: Definition, framework, and potential. *Quality and Quantity.* 2013;47(2):659-676.

82. Pluye P, Nha Hong Q. Combining the power of stories and the power of numbers: Mixed methods research and mixed studies reviews. *Annual Review of Public Health.* Vol 35: Annual Reviews Inc.; 2014:29-45.

83. Pluye P, Gagnon MP, Griffiths F, Johnson-Lafleur J. A scoring system for appraising mixed methods research, and concomitantly appraising qualitative, quantitative and mixed methods primary studies in Mixed Studies Reviews. *International Journal of Nursing Studies.* 2009;46(4):529-546.

84. New Zealand Mental Health Commission. *Blueprint for mental health services in New Zealand. How things need to be.* Wellington: Mental Health Commission;1998.

85. Anthony WA. Recovery from mental illness: The guiding vision of the mental health service system in the 1990s. *Psychosocial Rehabilitation Journal.* 1993;16(4):11-23.

86. Davidson L, Roe D. Recovery from versus recovery in serious mental illness: One strategy for lessening confusion plaguing recovery. *Journal of Mental Health.* 2007;16(4):459-470.

87. Richardson WS, Wilson MC, Nishikawa J, Hayward RS. The well-built clinical question: a key to evidence-based decisions. *ACP J Club.* 1995;123(3):A12-13.

88. Nilsen P. Making sense of implementation theories, models and frameworks. *Implementation Science.* 2015;10(1):53.

93. Munn Z, Tufanaru C, Aromataris E. Data extraction and synthesis. *American Journal of Nursing.* 2014;114(7):49-54.

94. Damschroder LJ, Aron DC, Keith RE, Kirsh SR, Alexander JA, Lowery JC. Fostering implementation of health services research findings into practice: a consolidated framework for advancing implementation science. *Implement Sci.* 2009;4:50.

95.

96. Aromataris E, Pearson A. The systematic review: an overview. *Am J Nurs.* 2014;114(3):53-58.

97. Kmet LM, Lee RC, Cook LS. *Standard quality assessment criteria for evaluating primary research papers from a variety of fields.* Alberta Heritage Foundation for Medical Research (AHFMR);2004. HTA Initiative #13.

For peer review only

For peer review only - http://bmjopen.bmj.com/site/about/guidelines.xhtml

98. Dixon-Woods M, Agarwal S, Jones D, Young B, Sutton A. Synthesising qualitative and quantitative evidence: A review of possible methods. *Journal of Health Services Research and Policy.* 2005;10(1):45-53.
99. Lucas PJ, Baird J, Arai L, Law C, Roberts HM. Worked examples of alternative methods for the synthesis of qualitative and quantitative research in systematic reviews. *BMC medical research methodology.* 2007;7.
100. Higgins JP, Altman DG, Gøtzsche PC, et al. The Cochrane Collaboration's tool for assessing risk of bias in randomised trials. *Bmj.* 2011;343:d5928.
101. Pope C, Mays N, Popay J. How can we synthesize qualitative and quantitative evidence for healthcare policy-makers and managers? *Healthcare Management Forum.* 2006;19(1):27-31.

For peer review only

For peer review only

For peer review only - http://bmjopen.bmj.com/site/about/guidelines.xhtml

APPENDIX 1 PRISMA-P 2015 Checklist

This checklist has been adapted for use with systematic review protocol submissions to BioMed Central journals from Table 3 in Moher D et al: Preferred reporting items for systematic review and meta-analysis protocols (PRISMA-P) 2015 statement. *Systematic Reviews* 2015 **4**:1

An Editorial from the Editors-in-Chief of *Systematic Reviews* details why this checklist was adapted - Moher D, Stewart L & Shekelle P: Implementing PRISMA-P: recommendations for prospective authors. *Systematic Reviews* 2016 **5**:15

Section/topic	#	Checklist item	Information reported Yes	Information reported No	Line number(s)
**ADMINISTRATIVE INFORMATION**					
**Title**					
Identification	1a	Identify the report as a protocol of a systematic review	☒	☐	1
Update	1b	If the protocol is for an update of a previous systematic review, identify as such	☐	☒	
**Registration**	2	If registered, provide the name of the registry (e.g., PROSPERO) and registration number in the Abstract	☐	☒	
**Authors**					
Contact	3a	Provide name, institutional affiliation, and e-mail address of all protocol authors; provide physical mailing address of corresponding author	☒	☐	5-34
Contributions	3b	Describe contributions of protocol authors and identify the guarantor of the review	☒	☐	278-280
**Amendments**	4	If the protocol represents an amendment of a previously completed or published protocol, identify as such and list changes; otherwise, state plan for documenting important protocol amendments	☐	☒	
**Support**					
Sources	5a	Indicate sources of financial or other support for the review	☒	☐	282
Sponsor	5b	Provide name for the review funder and/or sponsor	☒	☐	282
Role of sponsor/funder	5c	Describe roles of funder(s), sponsor(s), and/or institution(s), if any, in developing the protocol	☐	☒	
**INTRODUCTION**					
**Rationale**	6	Describe the rationale for the review in the context of what is already known	☒	☐	66-103

BioMed Central The Open Access Publisher

Section/topic	#	Checklist item	Information reported Yes	Information reported No	Line number(s)
**Objectives**	7	Provide an explicit statement of the question(s) the review will address with reference to participants, interventions, comparators, and outcomes (PICO)	☒	☐	104-116
**METHODS**					
**Eligibility criteria**	8	Specify the study characteristics (e.g., PICO, study design, setting, time frame) and report characteristics (e.g., years considered, language, publication status) to be used as criteria for eligibility for the review	☒	☐	119-151
**Information sources**	9	Describe all intended information sources (e.g., electronic databases, contact with study authors, trial registers, or other grey literature sources) with planned dates of coverage	☒	☐	153-165
**Search strategy**	10	Present draft of search strategy to be used for at least one electronic database, including planned limits, such that it could be repeated	☒	☐	166-175
STUDY RECORDS					
Data management	11a	Describe the mechanism(s) that will be used to manage records and data throughout the review	☒	☐	117-182
Selection process	11b	State the process that will be used for selecting studies (e.g., two independent reviewers) through each phase of the review (i.e., screening, eligibility, and inclusion in meta-analysis)	☒	☐	183-191
Data collection process	11c	Describe planned method of extracting data from reports (e.g., piloting forms, done independently, in duplicate), any processes for obtaining and confirming data from investigators	☒	☐	192-201
**Data items**	12	List and define all variables for which data will be sought (e.g., PICO items, funding sources), any pre-planned data assumptions and simplifications	☒	☐	203
**Outcomes and prioritization**	13	List and define all outcomes for which data will be sought, including prioritization of main and additional outcomes, with rationale	☒	☐	203
**Risk of bias in individual studies**	14	Describe anticipated methods for assessing risk of bias of individual studies, including whether this will be done at the outcome or study level, or both; state how this information will be used in data synthesis	☒	☐	216-217
DATA					
**Synthesis**	15a	Describe criteria under which study data will be quantitatively synthesized	☐	☒	
	15b	If data are appropriate for quantitative synthesis, describe planned summary measures, methods of handling data, and methods of combining data from studies, including any planned exploration of consistency (e.g., I^2, Kendall's tau)	☐	☒	

BioMed Central
The Open Access Publisher

For peer review only - http://bmjopen.bmj.com/site/about/guidelines.xhtml

Section/topic	#	Checklist item	Information reported		Line number(s)
			Yes	No	
	15c	Describe any proposed additional analyses (e.g., sensitivity or subgroup analyses, meta-regression)	☐	☒	
	15d	If quantitative synthesis is not appropriate, describe the type of summary planned	☒	☐	221-236
**Meta-bias(es)**	16	Specify any planned assessment of meta-bias(es) (e.g., publication bias across studies, selective reporting within studies)	☐	☒	
**Confidence in cumulative evidence**	17	Describe how the strength of the body of evidence will be assessed (e.g., GRADE)	☒	☐	205-219

For peer review only

BioMed Central The Open Access Publisher

APPENDIX 2

Pilot search strategy for Ovid MEDLINE(R) In-Process & Other Non-Indexed Citations, Ovid MEDLINE(R) Daily, Ovid MEDLINE(R) and Ovid OLDMEDLINE(R) 1946 to Present

#	Searches
1	recovery oriented.ti,ab,kw.
2	exp Mental Health Services/
3	recover*.ti,ab,kw.
4	2 and 3
5	Mental Health/
6	mental disorders/ or adjustment disorders/ or exp anxiety disorders/ or exp delirium, dementia, amnestic, cognitive disorders/ or exp dissociative disorders/ or exp mood disorders/ or exp neurotic disorders/ or exp personality disorders/ or exp "schizophrenia and disorders with psychotic features"/
7	((Mental or psychiatric or psychologic*) adj2 (health or illness* or disorder* or disease* or problem* or issue or issues or well being)).ti,ab,kw.
8	((adjustment or anxiety or obsessive or complusive or panic or phobic or stress or dissociative or mood or depressive or affective or personality or psychotic or paranoid) adj2 (disorder* or disease*)).ti,ab,kw.
9	(agoraphobi* or astheni* or phobia* or delirium or dementia* or depression or schizophreni* or schizoid).ti,ab,kw.
10	5 or 6 or 7 or 8 or 9
11	exp health services/ or service*.ti,ab,kw.
12	10 and 11
13	exp "Outcome and Process Assessment (Health Care)"/
14	exp delivery of health care/
15	Resource Allocation/
16	exp "Organization and Administration"/
17	exp "Quality of Health Care"/
18	exp professional competence/
19	exp Inservice Training/
20	exp patient care management/
21	((service* or organization* or organisation* or unit or units or department* or program* or

For peer review only

clinic or clinics or ward or wards or staff or workforce or setting) adj4 (leader* or innovate* or objective* or change* or transform* or structure* or opportunit* or strength* or model* or priorit* or policy or policies or procedure* or ration* or allocat* or reform* or commit* or needs or improve* or assess* or responsive* or evaluat* or plan*)).ti,ab,kw.

22 ((treatment or therapy) adj2 planning).ti,ab,kw.

23 13 or 14 or 15 or 16 or 17 or 18 or 19 or 20 or 21 or 22

24 exp Patient-Centered Care/

25 exp Self Care/ or exp Self Concept/

26 ((self or personal) adj2 (identity or concept or assess* or manage* or disclos* or efficacy or evaluat*)).ti,ab,kw.

27 ((patient or client or consumer*) adj4 (centered or centred or focus* or engage* or involve*)).ti,ab,kw.

28 ((shared or joint) adj2 decision*).ti,ab,kw.

29 exp Interpersonal Relations/

30 "Quality of Life"/ or quality of life.mp.

31 Social Support/ or Social Adjustment/

32 (social adj2 (support or integration or inclusion or adjustment)).ti,ab,kw.

33 (connected* or hope* or optimis* or trust or identity or meaning or empower* or spiritual* or giving back or stigma* or values or goal or goals or belonging or purpose or choice or citizenship or discriminat* or inequalit* or valued or roles).ti,ab,kw.

34 (partnership* or collaborat* or interpersonal or multidisciplinary or cooperat* or co-operat* or team* or group or joint).ti,ab,kw.

35 24 or 25 or 26 or 27 or 28 or 29 or 30 or 31 or 32 or 33 or 34

36 exp Data Collection/

37 exp Psychological Tests/

38 (measur* or test* or scale* or metric* or psychometric* oe questionnaire* or instrument* or data).ti,ab,kw.

39 36 or 37 or 38

40 23 or 39

41 12 and 40

42 41 and recover*.mp.

43 1 or 4 or 42

For peer review only

BMJ Open

Protocol for a mixed studies systematic review on the implementation of the recovery approach in adult mental health services

Journal:	*BMJ Open*
Manuscript ID	bmjopen-2017-017080.R2
Article Type:	Protocol
Date Submitted by the Author:	18-Jul-2017
Complete List of Authors:	Piat, Myra; McGill University, Psychiatry; Douglas Mental Health University Institute,<br>Sofouli, Eleni; Douglas Mental Health University Institute<br>Sabetti, Judith; McGill University, School of Social Work<br>Lambrou, Angella; McGill University<br>Chodos, Howard; Mental Health Commission of Canada - Ottawa<br>Briand, Catherine; Inst Univ Sante Mentale Montreal,<br>Vachon, Brigitte ; Universite de Montreal Faculte de medecine<br>Curran, Janet; Dalhousie University - Faculty of Health Professions
**Primary Subject Heading**:	Mental health
Secondary Subject Heading:	Evidence based practice, Health services research
Keywords:	MENTAL HEALTH, Adult psychiatry < PSYCHIATRY, Recovery, HEALTH SERVICES

SCHOLARONE™
Manuscripts

Protocol for a mixed studies systematic review on the implementation of the recovery approach in adult mental health services

Authors:

Myra Piat[1,2,3] ,PhD,
Eleni Sofouli[3] ,MSW,
Judith Sabetti[2,3] ,PhD,
Angella Lambrou[4] ,MLS
Howard Chodos, PhD
Catherine Briand [5], PhD,
Brigitte Vachon [5], PhD,
Janet Curran[6], PhD,

Author Affiliations:

[1]Department of Psychiatry, McGill University, Montreal, QC, Canada
[2]School of Social Work, McGill University, Montreal, QC, Canada
[3]Douglas Mental Health University Institute, Research Center, Montreal, QC, Canada
[4]Schulich Library of Physical Sciences, Life Sciences and Engineering, McGill University, QC, Canada (retired)
[5] Faculty of Medicine, University of Montreal, Montreal, QC, Canada
[6] School of Nursing, Faculty of Health Professions, Dalhousie University, Halifax, NS, Canada

Email:
myra.piat@douglas.mcgill.ca
eleni.sofouli@douglas.mcgill.ca
judith.sabetti@douglas.mcgill.ca
angella.lambrou@mcgill.ca
catharine.briand@umontreal.ca
brigitte.vachon@umontreal.ca
hchodos@rogers.com
janet.curran@iwk.nshealth.ca

Corresponding author: Myra Piat, PhD myra.piat@douglas.mcgill.ca

Word count: 3049

For peer review only - http://bmjopen.bmj.com/site/about/guidelines.xhtml

ABSTRACT

Introduction:

Recovery is integral to mental health planning in G-8 countries including Canada. A recovery-oriented approach to care aims to promote personal empowerment, illness self-management, and a life beyond services for people with serious mental illness (SMI), while reducing the financial burden associated with mental illness. Although there is a growing body of literature on recovery, no synthesis of research on the implementation of recovery into mental health services exists.

Objectives:

The objective is to conduct a mixed studies systematic review on the operationalization of recovery into mental health services for adults with SMI. It will inform the transformation of Canadian services to a recovery-orientation, but may be applicable to other countries.

Method and analysis:

Seven databases including PubMed, Ovid Medline, Ovid Embase, Ovid PsycInfo, CINAHL, the Cochrane Library and Scopus will be searched for peer-reviewed empirical studies published from 1998 to December 2016. Systematic reviews, and studies using quantitative, qualitative and mixed methodologies will be included. Secondary searches will be conducted in reference lists of all selected full text articles. Hand searches will also be performed in the tables of contents of three recovery-focused journals for the last 5 years. International experts in the field will be contacted for comments and advice. Data extraction will include identification and methodological synthesis of each study; definition of recovery; information on recovery implementation; facilitators and barriers; and study outcomes. A quality assessment will be conducted on each study. The data will be synthesized and a stepwise thematic analysis performed.

Ethics and dissemination:

Ethics approval is not required for this knowledge synthesis. Findings will be disseminated through knowledge translation activities including: a) a one-day symposium; b) presentations in national and international conferences, and to local stakeholders; c) publications in peer-reviewed journals, d) posts on the organizational websites.

For peer review only - http://bmjopen.bmj.com/site/about/guidelines.xhtml

Strengths and limitations of this study

- Studies included in this knowledge synthesis will have been conducted in inpatient, outpatient and community-based mental health settings, and will cover a broad range of research methodologies. The synthesis will reveal how recovery is understood; challenges involved in implementation; and, overall, to what extent transformation to recovery oriented services and systems is occurring.
- The selection of recovery-oriented studies with an implementation focus is unique, and will allow us to draw on one powerful conceptual models from implementation science that provide theory-informed elements to guide data analysis and synthesis as well as the reporting of results.
- Knowledge from the synthesis will be compiled into comprehensive and usable formats for organizational and government stakeholders, providing practical guidelines for recovery-based service reform and future evaluation.
- Limiting the search to published, peer-reviewed studies, while important for considerations of quality and methodological rigor, may overlook possible research on recovery-oriented services reported elsewhere.

INTRODUCTION

Rationale

Recovery is the focus of national mental health plans in G-8 countries[1-3]including Canada's first mental health strategy, *Changing Directions, Changing Lives*[4], and several provincial strategies[5-8]. The rationale for transformation to recovery-oriented services in mental health is compelling. While traditional mental health services have underlined professional control[9-13], reinforcing patient dependency, self-stigma and hopelessness[14-17], recovery approaches focus on individual empowerment, strong collaborative relationships between mental health service providers and service users, and community integration[18-24]. In promoting a life beyond services, recovery also meets a key ethical obligation to honor the personhood and citizenship of people with mental illness[25].

For peer review only - http://bmjopen.bmj.com/site/about/guidelines.xhtml

Recovery knowledge and evidence have burgeoned over the past two decades. Research exists on personal recovery[26-32], recovery-oriented services[33-40], and provider competencies[41-45]. Conceptual frameworks and standardized measures have been produced[35,46-49]. Other studies have linked the recovery approach to recognized theories, such as empowerment theory[50,51], the strengths model[52], capabilities theory[53-55], positive psychology[56-58], person-centered practice[59,60] and co-production[61,62]. Practice guidelines for recovery-oriented service provision are available[63-69], as well. In terms of the empirical literature, studies on particular agencies and programs have identified potential determinants of recovery orientation in services: for example a flexible and innovative organizational culture, results-oriented leadership and larger budgets[70] were found to be associated with recovery-oriented services, as were provider socio-professional characteristics such as greater age, higher educational levels and more professional experience[71,72]. Another recent study found that increasing the recovery orientation of teamwork on mental health teams[73] was associated with provider and consumer perceptions that services were recovery-oriented.

While two recent systematic reviews have been conducted on the recovery-oriented practices of mental health service providers[74,75], no known review has been published, to date, on the implementation of the recovery approaches into mental health services. Our review synthesizes research on the nature of recovery-oriented services, implementation challenges and overall system transformation. Work on the project was initiated in August 2016, and should come to completion in spring 2018.

This project is important and timely, as mental illness affects millions of people worldwide. According to recent World Health Organization statistics[76], 350 million people are

For peer review only - http://bmjopen.bmj.com/site/about/guidelines.xhtml

impacted by depression; 60 million people by bipolar disorder, and 21 million affected by schizophrenia. A recovery-oriented approach to healthcare is expected to reduce dependency and reduce the cost of mental healthcare, which in Canada has risen to more than $50 billion per year[4,77,78]. The project responds to a critical knowledge gap identified by knowledge users across Canada, who are responsible for implementing provincial level policy as well as shifting mental health organizations and services to a recovery orientation.

Objectives

The overall goal of this review is to systematically search, assess, and synthesize, implementation studies on mental health recovery from the international mental health literature in order to inform, and facilitate, the transformation of Canadian mental health systems and adult services to a recovery orientation. The following six research questions, guided by the Consolidated Framework for Advancing Implementation Science (CFIR)[79] will be applied to each selected study: (1) How was recovery defined in this study?; (2) How was the recovery approach implemented in this study (Intervention)? (3) What elements from the external environment (Outer setting), or internal environment (Inner setting), influenced implementation in this study?; (4) What were the characteristics of participants in this study? (Characteristics of individuals); (5) What processes were involved in effecting the implementation?; and 6) What was the extent and effectiveness of implementation in this study?

METHODS

Eligibility criteria

For peer review only - http://bmjopen.bmj.com/site/about/guidelines.xhtml

The design and methodology for the present review are reported following the Preferred Reporting Items for Systematic review and Meta-Analysis Protocols (PRISMA-P) guidelines[80] (Appendix 1).

Population: The review concerns studies of services for adults (18 yrs. +) with a primary diagnosis of schizophrenia, bipolar disorder or major depression, following the DSM-V classification for mental disorders.

Intervention: Studies will be included if they describe and evaluate the implementation of any intervention based on recovery principles that aims at transforming the orientation of mental health services, or organizations to a recovery approach.

Comparators: Studies will be eligible for inclusion whether or not they include comparison groups.

Outcomes: Selected studies should report outcomes related to the transformation of a mental health service, or organization, to a recovery-orientation. Outcomes might include change in organizational culture; more integrated service networks and partnerships; increased knowledge, skills and/or attitudinal change among mental health providers; more use of evidence based recovery-oriented best practices; greater consumer/provider collaboration, consumer self-management, and evaluation.

Study design: This will be a mixed studies review (MSR)[81,82]. The MSR integrates qualitative, quantitative and mixed methods studies, providing a rich, detailed understanding of complex health interventions and programs[83]. Studies representing a full range of methodologies will be included: systematic reviews and meta-analyses, randomized control trials (RCTs) and clinical trials, observational, mixed method and qualitative studies.

For peer review only - http://bmjopen.bmj.com/site/about/guidelines.xhtml

Time period: We would expect to find very few pertinent studies prior to 1998, when recovery was first defined in an international policy document[84]. Thus, the precise time frame for the review is 1998 to December 2016.

Setting: Research settings may include inpatient, outpatient or community-based mental health services.

Exclusion criteria: Non-research studies (e.g. editorials, letters, conference abstracts), as well as unpublished (grey) literature, dissertations, and book or book-length studies, will be excluded, as well as conceptual papers and review articles. Studies on services for addiction populations will also be excluded, as recovery is conceptualized differently in the addictions field. Language restrictions will not apply.

Information sources

Our final search strategy will be developed in consultation with an experienced research librarian on the project and will combine a broad, systematic search of the literature. Electronic search will be conducted on the following databases: PubMed, Ovid Medline, Ovid Embase, Ovid PsycInfo, CINAHL (Current Index to Nursing and Allied Health Literature), the Cochrane Library and Scopus. We will supplement our results by conducting the following secondary searches: (a) Reference lists of all selected full text articles will be scanned for additional relevant studies; (b) Citation-tracking will be performed on included articles; (c) Hand searches of tables of contents for the past 5 years will be conducted in the following key journals: *Psychiatric Rehabilitation Journal*; *Psychiatric Services*; and *Community Mental Health Journal*. Additional journals will be added if warranted; and (d) Known experts in the field will be

For peer review only

For peer review only - http://bmjopen.bmj.com/site/about/guidelines.xhtml

contacted for comments and advice. We will also stay alert to serendipitous discovery that may increase results.

Pilot Search strategy

Using the Ovid Medline database, a research librarian and co-investigator on the project conducted a pilot search (Appendix 2) which generated 5164 records. For this preliminary scoping phase, the search strategy was designed to focus on 3 main components: *mental health*, *recovery* and *services*. Medical Subject Heading (MeSH) and synonyms (keywords) were combined for each of the components. Terms related to recovery were chosen to reflect the consumer-survivor understanding of recovery[48,85,86] While keywords will remain consistent throughout the searches, subject headings will be revised to reflect database specific preferences. Search strategies will be further revised as new subject headings and keywords are revealed.

STUDY RECORDS

Data management

Electronic search results will be downloaded into EndNote reference manager software, duplicates will be removed where possible, and the remaining references will be uploaded to the Distiller Systematic Review software for the screening and data extraction stages. Distiller software stores references, manages and monitors the screening and data extraction process with customized forms and automated flowcharts, and provides an audit trail for the review.

Screening and Selection process

For the first selection, two team members working independently will read titles and abstracts of each paper identified in the electronic search and assess them for relevance based

For peer review only - http://bmjopen.bmj.com/site/about/guidelines.xhtml

on the inclusion and exclusion criteria. Second, the team members will read the full text of each selected article in order to confirm its inclusion in the study. Disagreements related to the inclusion of any paper will be discussed and resolved, involving a third team member if necessary. To ensure high inter-rater reliability, training exercises will be conducted prior to initiating the screening process. Team members will meet on a weekly basis to follow up on the screening process and discuss unanticipated problems.

Data items and data extraction process

In order to minimize bias, two research team members will independently extract the data. Sample elements for data extraction appear below in Table 1. The categories on the extraction grid include methodological elements based on the PICO mnemonic (PICO= population, intervention, comparison, outcome[87]). As well, elements corresponding to the 6 research questions will be extracted and organized using the CFIR[79], a multilevel five-dimension determinant framework[88] that constitutes a highly useful tool for identifying barriers and facilitators influencing implementation outcomes. Study limitations and gaps in knowledge will also be recorded[89]. The data extraction form will be pre-tested by the two reviewers and revised as needed. Distiller SR software will be used to manage the data extraction process.

Table 1: Sample elements for data extraction

For peer review only - http://bmjopen.bmj.com/site/about/guidelines.xhtml

Study ID/ Methods	Recovery Definition & Intervention	Characteristics of the Setting		Characteristics of Individuals	Process	Implementation Outcomes
		Outer	Inner			
I.D. #; location<br>Objective<br>Study design<br>Participants<br>Interventions/ comparisons<br>Data collection<br>Data analysis/ triangulation<br>Quality appraisal	-Recovery definition/ conceptualization<br><br>-Intervention Characteristics<br>source<br>adaptability<br>trialability<br>complexity<br>design& packaging<br>costs	-Needs & resources<br>-External links<br>-Political pressures<br>-Policy & incentives	-Structure<br>-Dialogue<br>-Culture<br>-Tension for change<br>-Values & Norms<br>-Priorities<br>-<br>Incentives & rewards	-Knowledge & beliefs<br>-Identification with organization<br>-Other personal attributes	-Advance planning<br>-« Buy-in »)<br>opinion leaders<br>implementation leaders<br>champions<br>-Execution<br>-Feedback	-extent of successful implementation of intervention<br><br>-Conclusions<br>-Study limitations/ gaps/<br>-Contradictions/ further questions

Quality assessment

Systematic reviews require that selected studies are assessed for quality[90]. We will use the Assessing Methodological Quality of Systematic Reviews tool (AMSTAR) protocol for the assessment of systematic reviews. The AMSTAR is an 11-item questionnaire that assesses study design, literature searched, and scientific quality of reviews; a rating system is included. For primary research studies, quality assessment will be determined using criteria developed by Kmet (2004)[91]. This tool includes a 14-item checklist for quality criteria in quantitative studies, and a 10-item checklist for qualitative studies. A rating system (yes-2; partial -1; and no-0) is provided, as well as a calculation for summary scores. While some controversy exists on whether qualitative research should be assessed using standard quality criteria[92], we will include a quality appraisal for qualitative, as well as quantitative, studies in order to better assess the strengths and weaknesses of the evidence[93]. The Cochrane Collaboration tool will be used for assessing the risk of bias in randomized control trials[94]. The selected studies will be

For peer review only

For peer review only - http://bmjopen.bmj.com/site/about/guidelines.xhtml

independently assessed for quality by two reviewers. Discrepancies will be solved in consultation with the principal investigator.

DATA

Data Synthesis

No single unifying framework exists for synthesizing quantitative and qualitative evidence for healthcare policy-makers and managers[95]. Our experience with recovery research suggests that much of the pertinent literature for review will be qualitative. Thus, our overall approach will be to convert all the evidence into qualitative form. The quantitative data will be transformed into qualitative form by extracting key concepts and findings within the elements geared to our research questions, as described above. Analytic procedures and synthesis will follow a 3-stage process: (a) organization of studies into logical categories according to their design, and methodology; and coding using NVivo 11 software; (b) within-study analysis, according to the study questions; (c) cross-study synthesis of the data using an adaptation of the stepwise thematic analysis developed by Lucas et al[93], according to the following procedures: (1) two reviewers will independently review data collated under each of the research questions; (2) codes produced by each researcher will be compared, and a consolidated list of themes produced for each research question; (3) themes occurring under each question will be clustered around common dimensions; (4) results of the thematic analysis will be presented to the research team at a consensus meeting.

Specific measures will be taken to enhance the trustworthiness of the data. As suggested by Lucas et al, directly reported participant data (e.g. verbatim quotations, or scores on attitudinal scales), and author interpretations, will be reported separately in order to retain

For peer review only - http://bmjopen.bmj.com/site/about/guidelines.xhtml

the richness or "thickness" of the data. Detailed descriptions, contextual material, and the quality assessment of each paper will also help readers make judgments about the reliability and validity of the data. Summary tables will include counts of the papers contributing data on each theme[92].

DISSEMINATION

Knowledge translation will involve collaboration with our international consultants, and knowledge users, who include decision makers and managers, service providers, people with lived experience and families. Four output documents will be developed, including: 1) a critical appraisal of findings from the synthesis on recovery implementation; 2) a compendium of case studies on successful recovery implementation initiatives (sensitive to gender, race, culture); 3) a recovery implementation manual for decision makers and managers; and 4) a toolkit of recovery-enhancing approaches, i.e. strategies for individual behavior change targeted at service providers and service users. Each document will be submitted to the entire team for revision and editing.

The results of the synthesis project will be widely disseminated. Knowledge translation activities will include: (a) creation of an Advisory Committee composed of the research team, Knowledge Users and international expert advisors. Quarterly telephone meetings with the Advisory to review emerging findings and provide feedback; (b) ongoing consultation/feedback between knowledge users and the research team during preparation of the 4 project outputs; (c) posting of information and updates on the websites of the organizations of knowledge users on the project, including those for people with lived experience and families; (d) organization of a one-day end-of-project symposium for dissemination of project outputs, including workshops

For peer review only - http://bmjopen.bmj.com/site/about/guidelines.xhtml

for recording feedback and recommendations; (e) dissemination of project outputs through organizational websites; and through national and international networks (free access); (f) submission of articles to peer-reviewed, open access journals; (g) presentations at national/international conferences;

CONCLUSION

The recovery approach emerged through the lived experiences of people with enduring mental health problems as they utilized the formal mental health care system. Recovery-oriented services are viewed as a more person-centered and promising approach for treating mental illness. Until now, there has been little access to knowledge concerning how mainstream mental health services are being transformed to a recovery-orientation, and with what results. Our synthesis will establish the state of knowledge and evidence on implementing recovery, and will make this knowledge available to a wide range of mental health stakeholders through dissemination activities and the publication of concrete recovery implementation tools. Results may also support the development of new recovery interventions, on which future outcome research should be considered.

Contributors:

Myra Piat (MP) is the guarantor. Myra Piat (MP), Eleni Sofouli (ES) and Judith Sabetti (JS) drafted the manuscript. Myra Piat (MP) Eleni Sofouli (ES), Judith Sabetti (JS), Catherine Briand (CB), Brigitte Vachon (BV) and Janet Curran (JC) contributed to the development of the selection criteria, and data extraction criteria. Angella Lambrou (AL) developed the preliminary search strategy. Howard Chodos (HC) Catherine Briand (CB), Brigitte Vachon (BV) and Janet Curran (JC) provided written feedback on the manuscript and Angella Lambrou (AL) Howard Chodos (HC) Catherine Briand (CB) Brigitte Vachon (BV) and Janet Curran (JC) approved the final manuscript.

For peer review only

For peer review only - http://bmjopen.bmj.com/site/about/guidelines.xhtml

Funding:

This study was funded by the Canadian Institute of Health Research, grant number KRS 144043.

Competing interests: None

REFERENCES

1. Adams N, Daniels A, Compagni A. International pathways to mental health transformation. *International Journal of Mental Health.* 2009;38(1):30-45.
2. Kirby M, Keon WJ. *Out of the shadows at last. Transforming mental health, mental illness and addiction services in Canada.* Ottawa: Sénat du Canada;2006.
3. Slade M, Williams J, Bird V, Leamy M, C L. Recovery grows up. *Journal of Mental Health.* 2012;21(2):99-103.
4. Mental Health Commission of Canada. *Changing directions, changing lives. The mental health strategy for Canada.* Calgary, AB2012.
5. Department of Health. The Action Plan for Mental Health in New Brunswick 2011-18. 2011:19.
6. Government of Manitoba. Rising to the challenge. A strategic plan for the mental health and well-being of Manitobans. 2011.
7. Ministère de la Santé et des Services sociaux (MSSS). *Plan d'action en santé mentale 2005-2010 : La force des liens.* Québec: MSSS;2005.
8. Piat M, Sabetti J. Recovery in Canada: Toward social equality. *International Review of Psychiatry.* 2012;24(1):19-28.
9. Ådnøy Eriksen K, Arman M, Davidson L, Sundfør B, Karlsson B. Challenges in relating to mental health professionals: Perspectives of persons with severe mental illness. *International Journal of Mental Health Nursing.* 2014;23(2):110-117.
10. Deegan PE. Recovery: The lived experience of rehabilitation. *Psychosocial Rehabilitation Journal.* 1988;11(4):11-19.
11. Gravel K, Légaré F, Graham ID. Barriers and facilitators to implementing shared decision-making in clinical practice: a systematic review of health professionals' perceptions. *Implementation Science.* 2006;1:16.
12. Légaré F, Ratte S, Gravel K, Graham ID. Barriers and facilitators to implementing shared decision-making in clinical practice: update of a systematic review of health professionals' perceptions. *Patient Education and Counseling.* 2008;73(3):526-535.
13. Nordt C, Rossler W, Lauber C. Attitudes of mental health professionals toward people with schizophrenia and major depression. *Schizophrenia Bulletin.* 2006;32(4):709-714.
14. Brohan E, Elgie R, Sartorious N, Thornicroft G. Self-stigma, empowerment and perceived discrimination among people with schizophrenia in 14 European countries: The GAMIAN-Europe study. *Schizophrenia Research.* 2010;122:232-238.
15. Corrigan PW, Watson AC, Barr L. The self-stigma of mental illness: Implications for self-esteem and self-efficacy. *Journal of Social and Clinical Psychology.* 2006;25(8):875-884.
16. Shahar G, Trower P, Iqbal Z, Birchwood M, Davidson L, Chadwick P. The person in recovery from acute and severe psychosis: the role of dependency, self-criticism, and efficacy. *American Journal of Orthopsychiatry.* 2004;74(4):480-488.
17. Watson AC, Corrigan P, Larson JE, Sells M. Self-stigma in people with mental illness. *Schizophrenia Bulletin.* 2007;33(6):1312-1318.
18. Davidson L, Chan KK. Common factors: evidence-based practice and recovery. *Psychiatric Services.* 2014;65(5):675-677.

For peer review only

For peer review only - http://bmjopen.bmj.com/site/about/guidelines.xhtml

19. Joosten EA, DeFuentes-Merillas L, de Weert GH, Sensky T, van der Staak CP, de Jong CA. Systematic review of the effects of shared decision-making on patient satisfaction, treatment adherence and health status. *Psychotherapy and Psychosomatics.* 2008;77(4):219-226.
20. Klockmo C, Marnetoft SU, Selander J, Nordenmark M. Important components to create personal working alliances with clients in the mental health sector to support the recovery process. *International Journal of Rehabilitation Research.* 2014;37(1):40-47.
21. Sweeney A, Fahmy S, Nolan F, et al. The relationship between therapeutic alliance and service user satisfaction in mental health inpatient wards and crisis house alternatives: A cross-sectional study. *PloS one.* 2014;9(7).
22. Borg M, Kristiansen K. Recovery-oriented professionals: Helping relationships in mental health services. *Journal of Mental Health.* 2004;13(5):493-505.
23. Crowe TP, Deane FP, Oades LG, Caputi P, Morland KG. Effectiveness of a collaborative recovery training program in Australia in promoting positive views about recovery. *Psychiatric Services.* 2006;57(10):1497-1500.
24. Green CA, Janoff SL, Yarborough BJ, Paulson RI. The recovery group project: development of an intervention led jointly by peer and professional counselors. *PsychiatrIC Services.* 2013;64(12):1211-1217.
25. Atterbury K. Preserving the person: The ethical imperative of recovery-oriented practices. *American Journal of Orthopsychiatry.* 2014;84(2):182-189.
26. Andresen R, Caputi P, Oades L. Stages of recovery instrument: development of a measure of recovery from serious mental illness. *Australian and New Zealand Journal of Psychiatry.* 2006;40(11-12):972-980.
27. Giffort D, Schmook A, Woody C, Vollendorf C, Gervain M. *Recovery Assessment Scale.* Chicago: Illinois Department of Mental Health;1995.
28. Khanam D, McDonald K, Williams Neils C. *Measuring recovery: A toolkit for mental health providers in New York City.* New York: The Bureau of Mental Health, NYC Department of Health and Mental Hygiene;2013.
29. Mueser KT, Gingerich S, Salyers MP, McGuire AB, Reyes RU, Cunningham H. *The Illness Management and Recovery (IMR) Scales (client and clinician versions).* Concord, N.H.: New Hampshire-Dartmouth Psychiatric Research Center;2004.
30. Neil ST, Kilbride M, Pitt L, et al. The questionnaire about the process of recovery (QPR): A measurement tool developed in collaboration with service users. *Psychosis.* 2009;1(2):145-155.
31. MacKeith J, Burns S. *Mental Health Recovery Star.* London: Mental Health Providers Forum and Triangle Consulting;2008.
32. Gordon SE, Ellis PM, Siegert RJ, Walkey FH. Core dimensions of recovery: A psychometric analysis. *Administration and Policy in Mental Health and Mental Health Services Research.* 2014;41(4):535-542.
33. Armstrong NP, Steffen JJ. The recovery promotion fidelity scale: assessing the organizational promotion of recovery. *Community Mental Health Journal.* 2009;45(3):163-170.
34. Bedregal LE, O'Connell M, Davidson L. The recovery knowledge inventory: Assessment of mental health staff knowledge and attitudes about recovery. *Psychiatric Rehabilitation Journal.* 2006;30(2):96-103.
35. Burgess P, Pirkis J, Coombs T, Rosen A. Assessing the value of existing recovery measures for routine use in Australian mental health services. *Australian and New Zealand Journal of Psychiatry.* 2011;45(4):267-280.
36. O'Connell M, Tondora J, Croog G, Evans A, Davidson L. From rhetoric to routine: assessing perceptions of recovery-oriented practices in a state mental health and addiction system. *Psychiatric Rehabilitation Journal.* 2005;28(4):378-386.

For peer review only - http://bmjopen.bmj.com/site/about/guidelines.xhtml

37. Onken SJ, Dumont JM, Ridgway P, Dornan DH, Ralph RO. Mental Health Recovery: What Helps and What Hinders? A National Research Project for the Development of Recovery Facilitating System Performance Indicators. Piloting the Recovery Oriented System Indicators (ROSI). 2006; http://www.familymentalhealthrecovery.org/conference/handouts/Workshop%208/Onken-ROSIPilotMeasuresV6.pdf. Accessed November 1, 2007.
38. Ridgway P, Press A. *Assessing the recovery-orientation of your mental health program. A User's guide for the Recovery-enhancing environment scale (REE).* Kansas: University of Kansas;2004.
39. Leamy M, Clarke E, Le Boutillier C, et al. Implementing a complex intervention to support personal recovery: A qualitative study nested within a cluster randomised controlled trial. *PloS one.* 2014;9(5).
40. Lloyd-Evans B, Mayo-Wilson E, Harrison B, et al. A systematic review and meta-analysis of randomised controlled trials of peer support for people with severe mental illness. *BMC Psychiatry.* 2014;14(1).
41. Russinova Z, Rogers ES, Ellison ML, Lyass A. Recovery-promoting professional competencies: perspectives of mental health consumers, consumer-providers and providers. *Psychiatric Rehabilitation Journal.* 2011;34(3):177-185.
42. National Institute for Mental Health in England. *Support, time and recovery (STR) workers. A competence framework.* London: Department of Health;2008.
43. National Social Inclusion Programme. *Capabilities for inclusive practice.* London: Department of Health;2007.
44. National Institute for Mental Health in England. *The ten essential shared capabilities. A framework for the whole of the mental health workforce.* London: Department of Health;2004. 40339.
45. New Zealand Ministry of Health. *Let's get real: Real skills for people working in mental health and addiction.* Wellington2008.
46. Leamy M, Bird V, Le Boutillier C, Williams J, Slade M. Conceptual framework for personal recovery in mental health: systematic review and narrative synthesis. *Br J Psychiatry.* 2011;199(6):445-452.
47. Williams J, Leamy M, Bird V, et al. Measures of the recovery orientation of mental health services: systematic review. *Soc Psychiatry Psychiatr Epidemiol.* 2012;47(11):1827-1835.
48. Slade M, Leamy M, Bacon F, et al. International differences in understanding recovery: Systematic review. *Epidemiology and psychiatric sciences.* 2012;21(4):353-364.
49. Bird V, Leamy M, Tew J, Le Boutillier C, Williams J, Slade M. Fit for purpose? Validation of a conceptual framework for personal recovery with current mental health consumers. *Australian and New Zealand Journal of Psychiatry.* 2014;48(7):644-653.
50. Rappaport J. Terms of empowerment/exemplars of prevention: Toward a theory for community psychology. *American Journal of Community Psychology.* 1987;15(2):121-148.
51. Zimmerman MA. Empowerment theory: Psychological, organizational and community levels of analysis. In: Rappaport J, Seidman E, eds. *Handbook of Community Psychology*. New York: Kluwer Academic/Plenum Publishers; 2000:43-63.
52. Rapp CA, Goscha RJ. *The Strengths Model: Case management with people with psychiatric disabilities.* Oxford, New Yord: Oxford University Press; 2006.
53. Burchardt T. Capabilities and disability: The capabilities framework and the social model of disability. *Disability and Society.* 2004;19(7):735-751.
54. Davidson L, Ridgway P, Wieland M, O'Connell M. A capabilities approach to mental health transformation: A conceptual framework for the recovery era. *Canadian journal of community mental health.* 2009;28(2):35-46.

For peer review only

For peer review only - http://bmjopen.bmj.com/site/about/guidelines.xhtml

55. Hopper K. Rethinking social recovery in schizophrenia: What a capabilities approach might offer. *Social Science and Medicine.* 2007;65(5):868-879.
56. Moran GS, Nemec PB. Walking on the sunny side: what positive psychology can contribute to psychiatric rehabilitation concepts and practice. *Psychiatric Rehabilitation Journal.* 2013;36(3):202-208.
57. Resnick SG, Rosenheck RA. Recovery and positive psychology: Parallel themes and potential synergies. *Psychiatric Services.* 2006;57(1):120-122.
58. Slade M. Mental illness and well-being: The central importance of positive psychology and recovery approaches. *BMC Health Services Research.* 2010;10.
59. Borg M, Karlsson B, Tondora J, Davidson L. Implementing person-centered care in psychiatric rehabilitation: what does this involve? *The Israel Journal of Psychiatry and Related Sciences.* 2009;46(2):84-93.
60. Rudnick A, Roe D. *Serious Mental Illness: Person-Centered Approaches.* London (GB): Radcliffe Publishing Ltd; 2011; 2014 Book Review.
61. Boyle D, Harris M. *The challenge of co-production. How equal partnerships between professionals and the public are crucial to improving public services.* London: NESTA;2009.
62. Denham-Vaughan S, Clark M. Care clusters in mental health and co-production of care-Towards a more lay friendly set of cluster descriptions. *Mental Health and Social Inclusion.* 2012;16(2):79-83.
63. Le Boutillier C, Leamy M, Bird VJ, Davidson L, Williams J, Slade M. What does recovery mean in practice? A qualitative analysis of international recovery-oriented practice guidance. *Psychiatric Services.* 2011;62(12):1470-1476.
64. Sowers W. Transforming systems of care: The American Association of Community Psychiatrists guidelines for recovery oriented services. *Community Mental Health Journal.* 2005;41(6):757-774.
65. Shepherd G, Boardman J, Slade M. *Making recovery a reality.* Sainsbury Centre for Mental Health;2008.
66. Shepherd G, Boardman J, Slade M. *Implementing recovery: A new framework for organisational change.* Sainsbury Centre for Mental Health;2009.
67. Australian Health Ministers' Advisory Council. *A national framework for recovery-oriented mental health services. Guide for practitioners and providers.* Canberra: Department of Health and Ageing;2013.
68. Newnham EA, Page AC. Bridging the gap between best evidence and best practice in mental health. *Clin Psychol Rev.* 2010;30(1):127-142.
69. Anthony WA, Mizock L. Evidence-Based Processes in an Era of Recovery: Implications for Rehabilitation Counseling and Research. *Rehabilitation Counseling Bulletin.* 2014;57(4):219-227.
70. Brown TT, Mahoney CB, Adams N, Felton M, Pareja C. What predicts recovery orientation in county departments of mental health? A pilot study. *Adm Policy Ment Health.* 2010;37(5):388-398.
71. McLoughlin KA, Fitzpatrick JJ. Self-reports of recovery-oriented practices of mental health nurses in state mental health institutes: development of a measure. *Issues in Mental Health Nursing.* 2008;29(10):1051-1065.
72. Osborn LA, Stein CH. Mental Health Care Providers' Views of Their Work with Consumers and Their Reports of Recovery-Orientation, Job Satisfaction, and Personal Growth. *Community Ment Health J.* 2015.
73. Leamy M, Clarke E, Le Boutillier C, et al. A national survey of recovery practice in community mental health teams. *British Journal of Psychiatry.* 2016;209(4):340-346.

For peer review only - http://bmjopen.bmj.com/site/about/guidelines.xhtml

74. Chester P, Ehrlich C, Warburton L, Baker D, Kendall E, Crompton D. “What is the work of Recovery Oriented Practice? A systematic literature review”. *International Journal of Mental Health Nursing.* 2016;25(4):270-285.

75. Le Boutillier C, Chevalier A, Lawrence V, et al. Staff understanding of recovery-orientated mental health practice: A systematic review and narrative synthesis. *Implementation Science.* 2015.

76. Mental disorders - Fact Sheet No. 396 [press release]. 2014.

77. Knapp M, Andrew A, McDaid D, et al. *Investing in recovery. Making the business case for effective interventions for people with schizophrenia and psychosis.* London, UK: Center for Mental Health;2014.

78. Latimer EA, Bond GR, Drake RE. Economic approaches to improving access to evidence-based and recovery-oriented services for people with severe mental illness. *Canadian Journal of Psychiatry.* 2011;56(9):523-529.

79. Damschroder LJ, Aron DC, Keith RE, Kirsh SR, Alexander JA, Lowery JC. Fostering implementation of health services research findings into practice: a consolidated framework for advancing implementation science. *Implementation Science.* 2009;4:50.

80. Shamseer L, Moher D, Clarke M, et al. Preferred reporting items for systematic review and meta-analysis protocols (prisma-p) 2015: Elaboration and explanation. *BMJ (Online).* 2015;349.

81. Heyvaert M, Maes B, Onghena P. Mixed methods research synthesis: Definition, framework, and potential. *Quality and Quantity.* 2013;47(2):659-676.

82. Pluye P, Nha Hong Q. Combining the power of stories and the power of numbers: Mixed methods research and mixed studies reviews. *Annual Review of Public Health.* Vol 35: Annual Reviews Inc.; 2014:29-45.

83. Pluye P, Gagnon MP, Griffiths F, Johnson-Lafleur J. A scoring system for appraising mixed methods research, and concomitantly appraising qualitative, quantitative and mixed methods primary studies in Mixed Studies Reviews. *International Journal of Nursing Studies.* 2009;46(4):529-546.

84. New Zealand Mental Health Commission. *Blueprint for mental health services in New Zealand. How things need to be.* Wellington: Mental Health Commission;1998.

85. Anthony WA. Recovery from mental illness: The guiding vision of the mental health service system in the 1990s. *Psychosocial Rehabilitation Journal.* 1993;16(4):11-23.

86. Davidson L, Roe D. Recovery from versus recovery in serious mental illness: One strategy for lessening confusion plaguing recovery. *Journal of Mental Health.* 2007;16(4):459-470.

87. Richardson WS, Wilson MC, Nishikawa J, Hayward RS. The well-built clinical question: a key to evidence-based decisions. *ACP J Club.* 1995;123(3):A12-13.

88. Nilsen P. Making sense of implementation theories, models and frameworks. *Implementation Science.* 2015;10(1):53.

89. Munn Z, Tufanaru C, Aromataris E. Data extraction and synthesis. *American Journal of Nursing.* 2014;114(7):49-54.

90. Aromataris E, Pearson A. The systematic review: an overview. *Am J Nurs.* 2014;114(3):53-58.

91. Kmet LM, Lee RC, Cook LS. *Standard quality assessment criteria for evaluating primary research papers from a variety of fields.* Alberta Heritage Foundation for Medical Research (AHFMR);2004. HTA Initiative #13.

92. Dixon-Woods M, Agarwal S, Jones D, Young B, Sutton A. Synthesising qualitative and quantitative evidence: A review of possible methods. *Journal of Health Services Research and Policy.* 2005;10(1):45-53.

93. Lucas PJ, Baird J, Arai L, Law C, Roberts HM. Worked examples of alternative methods for the synthesis of qualitative and quantitative research in systematic reviews. *BMC medical research methodology.* 2007;7.

For peer review only

For peer review only - http://bmjopen.bmj.com/site/about/guidelines.xhtml

94. Higgins JP, Altman DG, Gøtzsche PC, et al. The Cochrane Collaboration's tool for assessing risk of bias in randomised trials. *Bmj.* 2011;343:d5928.
95. Pope C, Mays N, Popay J. How can we synthesize qualitative and quantitative evidence for healthcare policy-makers and managers? *Healthcare Management Forum.* 2006;19(1):27-31.

For peer review only

For peer review only - http://bmjopen.bmj.com/site/about/guidelines.xhtml

APPENDIX 1 PRISMA-P 2015 Checklist

This checklist has been adapted for use with systematic review protocol submissions to BioMed Central journals from Table 3 in Moher D et al: Preferred reporting items for systematic review and meta-analysis protocols (PRISMA-P) 2015 statement. *Systematic Reviews* 2015 **4**:1

An Editorial from the Editors-in-Chief of *Systematic Reviews* details why this checklist was adapted - Moher D, Stewart L & Shekelle P: Implementing PRISMA-P: recommendations for prospective authors. *Systematic Reviews* 2016 **5**:15

Section/topic	#	Checklist item	Information reported		Line number(s)
			Yes	No	
**ADMINISTRATIVE INFORMATION**					
**Title**					
Identification	1a	Identify the report as a protocol of a systematic review	☒	☐	1
Update	1b	If the protocol is for an update of a previous systematic review, identify as such	☐	☒	
**Registration**	2	If registered, provide the name of the registry (e.g., PROSPERO) and registration number in the Abstract	☐	☒	
**Authors**					
Contact	3a	Provide name, institutional affiliation, and e-mail address of all protocol authors; provide physical mailing address of corresponding author	☒	☐	5-34
Contributions	3b	Describe contributions of protocol authors and identify the guarantor of the review	☒	☐	278-280
**Amendments**	4	If the protocol represents an amendment of a previously completed or published protocol, identify as such and list changes; otherwise, state plan for documenting important protocol amendments	☐	☒	
**Support**					
Sources	5a	Indicate sources of financial or other support for the review	☒	☐	282
Sponsor	5b	Provide name for the review funder and/or sponsor	☒	☐	282
Role of sponsor/funder	5c	Describe roles of funder(s), sponsor(s), and/or institution(s), if any, in developing the protocol	☐	☒	
**INTRODUCTION**					
**Rationale**	6	Describe the rationale for the review in the context of what is already known	☒	☐	66-103

BioMed Central The Open Access Publisher

Section/topic	#	Checklist item	Information reported Yes	Information reported No	Line number(s)
**Objectives**	7	Provide an explicit statement of the question(s) the review will address with reference to participants, interventions, comparators, and outcomes (PICO)	☒	☐	104-116
**METHODS**					
**Eligibility criteria**	8	Specify the study characteristics (e.g., PICO, study design, setting, time frame) and report characteristics (e.g., years considered, language, publication status) to be used as criteria for eligibility for the review	☒	☐	119-151
**Information sources**	9	Describe all intended information sources (e.g., electronic databases, contact with study authors, trial registers, or other grey literature sources) with planned dates of coverage	☒	☐	153-165
**Search strategy**	10	Present draft of search strategy to be used for at least one electronic database, including planned limits, such that it could be repeated	☒	☐	166-175
STUDY RECORDS					
Data management	11a	Describe the mechanism(s) that will be used to manage records and data throughout the review	☒	☐	117-182
Selection process	11b	State the process that will be used for selecting studies (e.g., two independent reviewers) through each phase of the review (i.e., screening, eligibility, and inclusion in meta-analysis)	☒	☐	183-191
Data collection process	11c	Describe planned method of extracting data from reports (e.g., piloting forms, done independently, in duplicate), any processes for obtaining and confirming data from investigators	☒	☐	192-201
**Data items**	12	List and define all variables for which data will be sought (e.g., PICO items, funding sources), any pre-planned data assumptions and simplifications	☒	☐	203
**Outcomes and prioritization**	13	List and define all outcomes for which data will be sought, including prioritization of main and additional outcomes, with rationale	☒	☐	203
**Risk of bias in individual studies**	14	Describe anticipated methods for assessing risk of bias of individual studies, including whether this will be done at the outcome or study level, or both; state how this information will be used in data synthesis	☒	☐	216-217
DATA					
**Synthesis**	15a	Describe criteria under which study data will be quantitatively synthesized	☐	☒	
	15b	If data are appropriate for quantitative synthesis, describe planned summary measures, methods of handling data, and methods of combining data from studies, including any planned exploration of consistency (e.g., I^2, Kendall's tau)	☐	☒	

BioMed Central
The Open Access Publisher

Section/topic	#	Checklist item	Information reported Yes	Information reported No	Line number(s)
	15c	Describe any proposed additional analyses (e.g., sensitivity or subgroup analyses, meta-regression)	☐	☒	
	15d	If quantitative synthesis is not appropriate, describe the type of summary planned	☒	☐	221-236
**Meta-bias(es)**	16	Specify any planned assessment of meta-bias(es) (e.g., publication bias across studies, selective reporting within studies)	☐	☒	
**Confidence in cumulative evidence**	17	Describe how the strength of the body of evidence will be assessed (e.g., GRADE)	☒	☐	205-219

For peer review only

BioMed Central
The Open Access Publisher

APPENDIX 2

Pilot search strategy for Ovid MEDLINE(R) In-Process & Other Non-Indexed Citations, Ovid MEDLINE(R) Daily, Ovid MEDLINE(R) and Ovid OLDMEDLINE(R) 1946 to Present

#	Searches
1	recovery oriented.ti,ab,kw.
2	exp Mental Health Services/
3	recover*.ti,ab,kw.
4	2 and 3
5	Mental Health/
6	mental disorders/ or adjustment disorders/ or exp anxiety disorders/ or exp delirium, dementia, amnestic, cognitive disorders/ or exp dissociative disorders/ or exp mood disorders/ or exp neurotic disorders/ or exp personality disorders/ or exp "schizophrenia and disorders with psychotic features"/
7	((Mental or psychiatric or psychologic*) adj2 (health or illness* or disorder* or disease* or problem* or issue or issues or well being)).ti,ab,kw.
8	((adjustment or anxiety or obsessive or complusive or panic or phobic or stress or dissociative or mood or depressive or affective or personality or psychotic or paranoid) adj2 (disorder* or disease*)).ti,ab,kw.
9	(agoraphobi* or astheni* or phobia* or delirium or dementia* or depression or schizophreni* or schizoid).ti,ab,kw.
10	5 or 6 or 7 or 8 or 9
11	exp health services/ or service*.ti,ab,kw.
12	10 and 11
13	exp "Outcome and Process Assessment (Health Care)"/
14	exp delivery of health care/
15	Resource Allocation/
16	exp "Organization and Administration"/
17	exp "Quality of Health Care"/
18	exp professional competence/
19	exp Inservice Training/
20	exp patient care management/
21	((service* or organization* or organisation* or unit or units or department* or program* or

For peer review only

clinic or clinics or ward or wards or staff or workforce or setting) adj4 (leader* or innovate* or objective* or change* or transform* or structure* or opportunit* or strength* or model* or priorit* or policy or policies or procedure* or ration* or allocat* or reform* or commit* or needs or improve* or assess* or responsive* or evaluat* or plan*)).ti,ab,kw.

22 ((treatment or therapy) adj2 planning).ti,ab,kw.

23 13 or 14 or 15 or 16 or 17 or 18 or 19 or 20 or 21 or 22

24 exp Patient-Centered Care/

25 exp Self Care/ or exp Self Concept/

26 ((self or personal) adj2 (identity or concept or assess* or manage* or disclos* or efficacy or evaluat*)).ti,ab,kw.

27 ((patient or client or consumer*) adj4 (centered or centred or focus* or engage* or involve*)).ti,ab,kw.

28 ((shared or joint) adj2 decision*).ti,ab,kw.

29 exp Interpersonal Relations/

30 "Quality of Life"/ or quality of life.mp.

31 Social Support/ or Social Adjustment/

32 (social adj2 (support or integration or inclusion or adjustment)).ti,ab,kw.

33 (connected* or hope* or optimis* or trust or identity or meaning or empower* or spiritual* or giving back or stigma* or values or goal or goals or belonging or purpose or choice or citizenship or discriminat* or inequalit* or valued or roles).ti,ab,kw.

34 (partnership* or collaborat* or interpersonal or multidisciplinary or cooperat* or co-operat* or team* or group or joint).ti,ab,kw.

35 24 or 25 or 26 or 27 or 28 or 29 or 30 or 31 or 32 or 33 or 34

36 exp Data Collection/

37 exp Psychological Tests/

38 (measur* or test* or scale* or metric* or psychometric* oe questionnaire* or instrument* or data).ti,ab,kw.

39 36 or 37 or 38

40 23 or 39

41 12 and 40

42 41 and recover*.mp.

43 1 or 4 or 42

For peer review only
